# Crystal Structure, Raman, FTIR, UV-Vis Absorption, Photoluminescence Spectroscopy, TG–DSC and Dielectric Properties of New Semiorganic Crystals of 2-Methylbenzimidazolium Perchlorate

**DOI:** 10.3390/ma16051994

**Published:** 2023-02-28

**Authors:** Elena Balashova, Andrey Zolotarev, Aleksandr A. Levin, Valery Davydov, Sergey Pavlov, Alexander Smirnov, Anatoly Starukhin, Boris Krichevtsov, Hongjun Zhang, Fangzhe Li, Huijiadai Luo, Hua Ke

**Affiliations:** 1 Ioffe Institute, Politechnicheskaya 26, 194021 Saint Petersburg, Russia; 2 Institute of Earth Sciences, Saint Petersburg State University, Universitetskaya Nab. 7/9, 199034 Saint Petersburg, Russia; 3 School of Instrument Science and Engineering, Harbin Institute of Technology, Harbin 150080, China; 4 School of Materials Sciences and Engineering, Harbin Institute of Technology, Harbin 150080, China

**Keywords:** semi-organic crystals, single crystal and powder XRD, Raman scattering, UV-Vis absorption, photoluminescence, phase transitions, dielectric properties

## Abstract

Single crystals of 2-methylbenzimidazolium perchlorate were prepared for the first time with a slow evaporation method from an aqueous solution of a mixture of 2-methylbenzimidazole (MBI) crystals and perchloric acid HClO_4_. The crystal structure was determined by single crystal X-ray diffraction (XRD) and confirmed by XRD of powder. Angle-resolved polarized Raman and Fourier-transform infrared (FTIR) absorption spectra of crystals consist of lines caused by molecular vibrations in MBI molecule and ClO_4_^−^ tetrahedron in the region *ν* = 200–3500 cm^−1^ and lattice vibrations in the region of 0–200 cm^−1^. Both XRD and Raman spectroscopy show a protonation of MBI molecule in the crystal. An analysis of ultraviolet-visible (UV-Vis) absorption spectra gives an estimation of an optical gap *E_g_*~3.9 eV in the crystals studied. Photoluminescence spectra of MBI-perchlorate crystals consist of a number of overlapping bands with the main maximum at *E_photon_* ≅ 2.0 eV. Thermogravimetry-differential scanning calorimetry (TG-DSC) revealed the presence of two first-order phase transitions with different temperature hysteresis at temperatures above room temperature. The higher temperature transition corresponds to the melting temperature. Both phase transitions are accompanied by a strong increase in the permittivity and conductivity, especially during melting, which is similar to the effect of an ionic liquid.

## 1. Introduction

At present, the search and study of new organic and semi-organic crystals is of great interest, which is associated with the need to get new multifunctional materials for energy harvesting [1], the creation of flexible materials for electronics [2,3], sensors of various fields, piezoelectric elements, nonlinear optical devices [4,5,6], fuel cell membranes for hydrogen energy [7], biomedical and biotechnological devices [8], etc. Particular attention is paid to ferroelectrics and related materials since, due to the absence of an inversion center in the crystal structure, they exhibit a combination of various physical properties (ferroelectricity, piezoelectric effect, pyroelectric effect, second-order nonlinear optical effects, etc.). Also of interest are new centrosymmetric organic crystals, since they, as a rule, have a wide transparency window and, at the same time, can exhibit significant third-order optical and dielectric nonlinear susceptibilities.

For the synthesis of new organic or semi-organic crystals, methods based on slow cooling or evaporation from various solutions are widely used since most organic and some inorganic materials dissolve in water, ethanol, acetone, etc. The number of new crystals synthesized by these methods increases greatly every year. Among them, we can mention the discovery of ferroelectricity in one-component organic crystals of croconic acid (C_5_O_5_H_2_, *P_s_*~30 μC/cm^2^ at room temperature (RT)) [9,10], 2-methylbenzimidazole (MBI, C_8_H_8_N_2_, *P_s_*~5–7 μC/cm^2^) [11] and two-component crystals of diisopropylammonium iodide (*P_s_*~33 μC/cm^2^) [12]. We also note that semi-organic crystals synthesized from solutions based on a combination of amino acids, such as glycine, betaine and inorganic acids, such as sulfuric, phosphorous and phosphoric acids, can exhibit ferroelectric properties (glycine phosphite [13], triglycine sulfate [14], betaine phosphite [15,16]) or antiferroelectric properties [17]. In deuterated crystals of amino acid ferroelectrics, the Curie temperature can be higher than room temperature [15,18].

It was known that the binding of molecules containing an imidazole ring (for example, histidine) with inorganic acids can lead to the formation of crystals with a non-centrosymmetric structure [19,20]. This aroused interest in the search for new crystals based on such combinations. In Ref. [21], crystals based on organic 2-methylbenzimidazole consisting of linked benzene and imidazole rings and inorganic phosphorous H_3_PO_3_ or phosphoric H_3_PO_4_ acids were synthesized and studied. X-ray diffraction (XRD) and Raman spectroscopy measurements have shown that the crystal structures of new substances were formed by the cations (MBI+H)^+^, anions H_2_PO_3_^−^ or H_2_PO_4_^−^ and water molecules. The crystal structure of MBI-phosphite is characterized by a centrosymmetric space group *P*2_1_/*c* (14) of monoclinic syngony, whereas the structures of MBI-phosphate-1 and the MBI-phosphate-2 modifications are described in the frame of the space group *P*$\bar{1}\;\left(2\right)$ of triclinic syngony. Dielectric measurements have shown that, in MBI-phosphite, an increase in temperature leads to a very strong increase in conductivity and the appearance of a specific behavior characteristic of proton superionics while in MBI phosphates, a decrease in temperature is accompanied by an increase in the dielectric constant, similarly to quantum paraelectric at low temperatures [21].

Perchlorate salts are of great interest for chemistry because of their unique properties such as high degree of ionic character (electronegativity), high solubility in different solvents, small possibility of oxidation or reduction, etc. [22]. They have been used for more than 50 years as a medicine for the treatment of thyroid gland disorders and are widely used in the pyrotechnics industry, and ammonium perchlorate is also a component of solid rocket fuel [23]. At present, perchlorate salts are also of interest for astrobiology. It has recently been found that perchlorate salts, which are ubiquitous on Mars, increase the activity of α-chymotrypsin at low temperatures, and the effect of perchlorate salts on the thermodynamics of α-chymotrypsin activity closely resembles psychrophilic adaptations [24,25].

Particular attention last time is paid to semi-organic crystals synthesized from aqueous solutions of perchloric acid HClO_4_ with various organic components. Many of these compounds exhibit ferroelectric [26,27,28] and nonlinear optical properties [29,30]. Among them are imidazole perchlorate crystals and films, characterized at RT by the polar space group *R*3*m* (160) of trigonal syngony and having a spontaneous polarization *P_s_*~6–8 μC/cm^2^ and a coercive field *E_c_*~1 kV/cm [22,24]. Note that the imidazole (Im) single crystals C_3_N_2_H_4_ are centrosymmetric (the space group *P*2_1_/*c* (14)) [31]. Nevertheless, an interaction between Im and perchloric acid molecules in solutions leads to the formation of a non-centrosymmetric imidazole perchlorate crystal. Unlike imidazole, MBI crystals are non-centrosymmetric and exhibit biaxial ferroelectricity above room temperature [11]. Because the crystals formed by perchloric acid with organic components show different phase transformation, it was interesting to know what structure the crystals formed by the combination of MBI and perchloric acid would have.

The main objectives of this work are the synthesis of a new salt of perchloric acid, 2-methylbenzimidazolium perchlorate, the study of its crystal structure, optical and dielectric properties and phase transitions. Studies of the crystal structure by means of single crystal and powder X-ray diffraction (XRD) are confirmed by the analysis of Raman and Fourier-transform infrared (FTIR) spectroscopy. The optical properties were studied using absorption spectroscopy in the ultraviolet-visible (UV-Vis) region and photoluminescence. Thermogravimetry-differential scanning calorimetry (TG-DSC) and dielectric measurements of new crystals and their analysis are presented.

## 2. Materials and Methods

### 2.1. Crystal Synthesis

**Figure 1:**
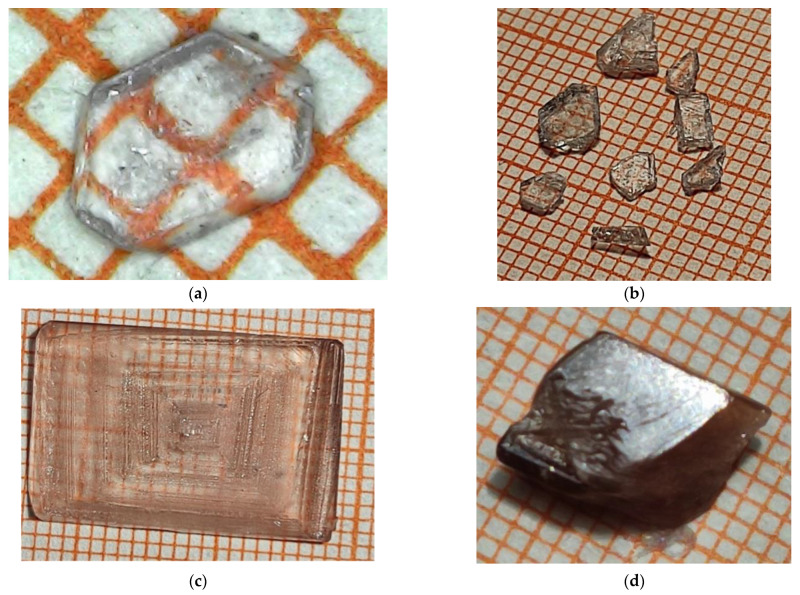
Optical images of grown MBI-perchlorate crystals. Samples 1 (**a**,**b**), 2 (**c**), and 3 (**d**). The sizes of the grid steps in (**a**–**d**) are equal to 1 mm.

Single crystals of 2-methylbenzimidazolium perchlorate (MBI-perchlorate or MBI⋅HClO_4_ or (C_8_H_8_N_2_) (HClO_4_)) were grown from aqueous solutions of MBI single crystals and perchloric acid by evaporation. MBI crystals used for synthesis were grown from chemically prepared MBI powder. To remove impurities, the powder was recrystallized several times in ethanol [32]. As a result, almost colorless MBI crystals were obtained. For the synthesis of MBI⋅HClO_4_, the purified MBI crystals were dissolved in ~70% aqueous solution of perchloric acid until almost all saturated solutions were obtained, and MBI⋅HClO_4_ crystals were grown from them at RT. Optical images of some synthesized crystals are shown in Figure 1.

MBI-perchlorate thin plates (Figure 1a,b (Sample 1)) are transparent and colorless. More thick single crystal shown in Figure 1c (Sample 2) has a faint pink color. The thick bulk crystal shown in Figure 1d (Sample 3) has a distinct brown tint indicating the presence of impurities caused most probably by the partial decomposition of perchloric acid over time in the solution, from which colorless crystals first grew. Therefore, the last crystals obtained from the solution were brown. The presence of impurities in the brown bulk crystal (Sample 3) is also confirmed by experiments with photoluminescence (see below). All crystals have a pronounced cut. The crystals are well dissolved in water or ethanol.

### 2.2. Crystal Structure Measurements

Crystal structure of MBI-perchlorate samples was investigated using single crystal and powder XRD analysis.

#### 2.2.1. Experimental Details of Single Crystal XRD

For single crystal XRD experiment, the suitable crystals of MBI-perchlorate (3 samples) were fixed on a micro mount and placed on at Rigaku XtaLAB Synergy and SuperNova diffractometers and were measured at a temperature of 100 K using monochromatized Cu-*K_α_* radiation (generated at a high voltage of 50 kV and a current of 1 mA of an X-ray tube with a copper anode) in the Resource Center “X-ray Diffraction Methods” of St. Petersburg State University. The structures have been solved by the direct methods by means of the *SHELX* program [33] incorporated in the *OLEX2* program package [34]. The non-hydrogen atoms were refined in an anisotropic approximation of atomic temperature factors. Equivalent isotropic factors *U*_eq_ were also calculated for these atoms ( see Section S.II of the Supplementary Materials). The carbon and nitrogen-bound H atoms were placed in calculated positions and were included in the refinement in the ‘riding’ model approximation with the isotropic temperature factors *U*_iso_(H) set to 1.5*U*_eq_(C) and C–H interatomic distances 0.96 Å (for sample 1) or 0.98 Å (for samples 2–3) for CH_3_ groups, with *U_iso_*(H) set to 1.2*U*_eq_(*C*) and C–H 0.93 Å (for sample 1) or 0.95 Å (for samples 2 and 3) for CH groups and with *U*_iso_(H) set to 1.2*U*_eq_(N) and N–H 0.86 Å (for sample 1) or 0.88 Å (for samples 2 and 3) for NH-groups. Empirical absorption correction was applied in *CrysAlisPro* program complex [CrysAlisPro, Agilent Technologies, Versions 1.171.40.50a (for sample 1) and 1.171.41.104a (for samples 2 and 3)] using spherical harmonics, implemented in *SCALE3 ABSPACK* scaling algorithm. The drawings of the MBI-perchlorate crystal structure were made using the *Vesta* program [35].

#### 2.2.2. Experimental Details of Powder XRD

In order to obtain evidence that the synthesized crystals consist at RT of the same phase of MBI-perchlorate, which was determined in the XRD experiment with single crystals at 100 K, as well as to obtain accurate values of their unit cell parameters and characterization of their microstructure on a nanometer scale, the powder MBI-perchlorate was also examined by XRD.

The powder of MBI-perchlorate was obtained by thoroughly grounding the synthesized crystals in a corundum mortar. The prepared powder was placed into the low-background single crystal Si(119) sample holder.

Powder X-ray diffractometer D2 PHASER (Bruker AXS, Karlsruhe, Germany) in vertical Bragg-Brentano geometry equipped with an X-ray tube with a copper anode, Ni filter (Cu-*K*_α_ radiation, wave length λ = 1.54184 Ǻ) and a position-sensitive semiconductor linear X-ray detector LYNXEYE was used for measurements of XRD powder pattern (2*θ*-*θ* scan mode, scan range 2*θ* = 5.6–120°, step Δ2*θ* = 0.02°). During the XRD measurements, the temperature *T*_meas_ in the sample chamber of the diffractometer was equal to 313 ± 1 K. Other details of measurements and obtaining the diffraction angle corrections (zero shift Δ2*θ*_0_ and displacement Δ2*θ*_displ_) of the XRD pattern are the same as described in [21,32,36,37] and are briefly given in Supplementary Materials (Section S.I).

First, the analysis of the measured XRD pattern was carried out utilizing program *EVA* [38], which determines the XRD reflection parameters (Miller indices *hkl*, Bragg angles 2*θ*_B_^obs^, full width at half maximum (FWHM) *FWHM*, maximum (*I*_max_) and integral (*I*_int_) intensities) used for preliminary calculation of the unit cell parameters using the crystallographic-oriented least squares program *Celsiz* [39] and XRD line profile analysis (LPA). To evaluate the microstructure parameters (mean crystallite sizes *D*, i.e., the sizes of the areas of the coherent scattering of X-rays in the crystals, and absolute values of mean microstrains *ε*_s_ in the crystallites), LPA was performed using Williamson-Hall plot (WHP) [40] and size-strain plot (SSP) [41] graphical methods implemented in the *SizeCr* [42] program for the pseudo-Voigt (pV) reflections [43] observed in the XRD pattern. The *SizeCr* program and WHP and SSP techniques are described in detail in original works [32,36,37] and in our previous paper [21]. Some details are given in Supplementary Materials (Section S.I). It should be emphasized that, often, the average size of crystallites is calculated either with the Scherrer equation using only one XRD reflection or with the original WHP method for Lorentz-type XRD reflections (see, for example, [44]). On the contrary, in the current investigation, the average size *D* of the crystallites and the absolute average value of the microstrain *ε*_s_ were estimated using the WHP and SSP methods implemented in the *SizeCr* program, modified for the observed pV type of XRD reflection profiles, taking into account all individual XRD reflections that could be extracted from the observed superimposed reflections.

The unit cell parameters and crystallite sizes (LPA showed the absence of microstrains, *ε*_s_ = 0), determined using the *Celsiz* and *SizeCr* programs at the first stage of research, were used as initial values for Le Bail (LB) [45] whole powder fitting the simulated XRD pattern to experimental one. The LB method allows for fitting without a structure and preferred orientation models when specifying only the space group of the compound, resulting in precision values of the unit cell parameters (*a*, *b*, *c* and *ß* for monoclinic cell), crystallite sizes *D* and, with good quality of fitting, giving proof of the single-phase powder.

In turn, the values of the parameters *a*, *b*, *c* and *ß* and *D* obtained in the LB fitting were used as the initial ones to refine the MBI⋅HClO_4_ structure and fit the model XRD pattern to the experimental one with the Rietveld method [46]. The starting atomic coordinates of Sample 1 (Table S1 of Supplementary Materials) were used in the Rietveld refinement.

Both LB and Rietveld refinement and fitting were carried out using the *TOPAS* program [42]. To evaluate the crystallite size *D* parameter, the pV reflection profiles were described from the first principles (FP, fundamental parameters approach) [47,48]. The same coefficient of the Scherrer equation as in the *SizeCr* program (*K*_Scherrer_ = 0.94 [42]) was used to calculate the crystal size in the *TOPAS* program from the simulated FWHM of the reflections using double Voigt’s approach [49].

The preferred orientation effects were corrected using March-Dollase (MD) approach [50] for two crystallographic directions ([101] and [111]) in accordance with the tool in *TOPAS*. Model of spherical harmonics of 8th order [51] was utilized for correction of the contribution of other directions of preferred orientation. Other details (weight scheme, emission spectrum of the Cu-*K*_α_ radiation, restrictions on bond length during the refinement of the atomic coordinates, use of the overall isotropic temperature factors *U*_iso_^overall^ for every sort of atoms, calculation of factor correcting the estimated standard deviations (e.s.d.s) obtained in LB and Rietveld refinement and checking and calculation of agreement factors) [52,53,54,55] and the course of the Rietveld refinement are generally the same as described in [21,36,37]. Some specific details are briefly described in Supplementary Materials (Section S.I).

### 2.3. Mass Density

The theoretical mass density (XRD mass density) of the compound in XRD experiments with single crystal and powder was taken from the calculations of the corresponding structure-refinement programs (*SCHELX* and *TOPAS*, respectively). This value is easily calculated according to relation (see [44] as an example).

(1) $$\rho_{\mathrm{calc}}\left(g/{\mathrm{cm}}^{3}\right)=\frac{M_{\mathrm{cell}}}{N_{A\;}\cdot V_{\mathrm{cell}}\cdot 10^{-24}}$$

where *M*_cell_ = *Z*∙*M*_mol_ is the mass of the unit cell (in g/mol), *Z* is the number of formula units in the unit cell (*Z* = 4 for MBI⋅HClO_4_), and *M*_mol_ is the molar mass of the formula unit, *V*_cell_ is the unit cell volume (in Å^3^), and *N*_A_ = 6.02214076⋅10^23^ mol^−1^ is the Avogadro constant.

The experimental value of the mass density of a single crystal was determined with the method of helium pycnometry at RT. In this method, based on the Archimedes principle, helium penetrates into the sample open pores with sizes of ~1 Å and larger, the volume of the displaced helium is measured, which is equal to the volume of the sample *V*_s_ (i.e., it is the sample geometrical volume minus the volume of these pores, which are filled with helium). The pycnometric density is calculated as *ρ*_pycn_ = *m*_s_/*V*_s_, where *m*_s_ is the sample mass (obtained by weighing). For the measurements of single crystal pycnometric density, an AccuPyc 1330 pycnometer (Micromeritics Instrument Corporation, Norcross, GA, USA) was used.

From the values of theoretical mass density *ρ*_calc_ and pycnometric density *ρ*_pycn_ obtained by measurements of powder XRD and pycnometry at close temperatures (*T*_meas_ = 313 K and RT, respectively), the compactness *C* and porosity *P* of the MBI⋅HClO4 crystal were determined in accordance with the expressions [44].

(2) $$C\left(\%\right)=\frac{\rho_{\mathrm{pycn}}}{\rho_{\mathrm{calc}}}\cdot 100\%$$

and

(3) $$P\left(\%\right)=\left(1-\frac{\rho_{\mathrm{pycn}}}{\rho_{\mathrm{calc}}}\right)\cdot 100\%.$$

### 2.4. Raman Scattering, FTIR, UV-Vis Absorption, and Photoluminescence

Raman spectra of the MBI-perchlorate single crystals were measured using a LabRAM HREvo UV-VIS-NIR open spectrometer (Horiba, Lille, France) equipped with a confocal microscope and a silicon CCD matrix cooled to the liquid nitrogen temperature. The line at *λ* = 532 nm (2.33 eV) of stabilized single longitudinal mode diode-pumped solid-state (DPSS) laser (Oxxius, Lannion, France) was used as the excitation source. The laser beam was focused using an Olympus 100× (*NA* = 0.90) objective lens onto a spot of diameter ~1 μm on the sample surface. To avoid laser-induced sample damage, the laser power on the samples was as low as ~25–80 µW. We used 1800 grooves/mm diffraction grating and 100× (*NA* = 0.90) objective lens to measure Raman spectra. In the low frequency spectral region, the Rayleigh line was suppressed using three BragGrate notch filters (OptiGrate Corp., Oviedo, FL, USA) with an OD = 4 and a spectral bandwidth < 10 cm^−1^. Polarized micro-Raman measurements were performed at RT (293 K), in the spectral range 5–4000 cm^−^^1^ at different scattering geometries. The backscattering geometries are given in Porto’s notation, for example, Z(XY)$\bar{Z}$. Here, Z-axis is oriented normally to the crystal surface (100), and X and Y are along c*- and b-crystal axes, respectively.

Angle-resolved polarized Raman spectroscopy is a powerful tool to diagnose anisotropic materials. Angle-resolved polarized Raman measurements were performed by incorporating an automatically rotating *λ*/2 wave plate directly in front of the objective to simultaneously vary the polarization directions of the incident laser beam and scattered Raman light. With this configuration, when passing through the wave plate, the polarization of the incident beam rotates in a controlled manner relative to the crystallographic axes of the immobile test sample. By selecting the appropriate polarization of scattered light transmitted through the same *λ*/2 wave plate with an analyzer placed in front of the slit of the spectrometer, it is possible to measure polarized Raman spectra depending on the angle between the polarization of the incident radiation and the crystallographic axes of the sample.

The micro-photoluminescence (µ-PL) measurements were carried out in microscope stage Linkam THMS600 (Linkam Sci. Inst. Ltd., Salfords, Surrey, UK). The line at *λ* = 266 nm (4.66 eV) of MPL-F-266-10 laser (CNI Laser, Changchun, China) was used for continuous wave (CW) excitation. We used 600 lines/mm grating and a large working distance lens (Mitutoyo 50× UV (*NA* = 0.40)) with a spot size of ~2 µm and power density of 6 kWcm^−^^2^ on a sample was used to measure µ-PL.

Measurements of the infrared (IR) absorption spectra (in FTIR) were carried out using an IR-Fourier spectrophotometer IRPrestige-21 with an IR microscope AIM-8000 (Shimadzu Corp., Kyoto, Japan), both in the specular reflection mode and in the transmission mode, followed by the Kramers–Kronig transformation. The results were then converted to absorbance. The measured spectral range was from 650 to 5000 cm^−1^.

Optical absorption spectra of MBI-perchlorate in the UV-Vis spectral range were obtained using a UV-3600i Plus UV-Vis-NIR spectrophotometer (Shimadzu Corp., Kyoto, Japan) operating at RT in the wavelength range of 200–2000 nm. The test was conducted in reflection mode using an integrating sphere. BaSO_4_ was used as a reference sample. Absorption spectra in aqueous solutions of MBI and HClO_4_ were measured on an SP-2000 spectrophotometer (OKB Spektr LLC, St. Petersburg, Russia) in the wavelength range of 200–1000 nm.

### 2.5. TG–DSC

TG–DSC studies were carried out in air using Thermal Analysis System: Mettler Toledo TGA/DSC 3+ (Mettler-Toledo, LLC, Columbus, OH, USA). Experiments were performed in the temperature interval 35–400 °C with different rates of heating and cooling (20 °C/min, 10 °C/min and 5 °C/min for crystals shown in Figure 1b (Sample 1)). Maximal temperature of heating–cooling cycles were 200 °C and 400 °C. During measurements, the samples were photographed.

### 2.6. Dielectric Measurements

Measurements of capacity and dielectric losses (tgδ) in single crystals of MBI-perchlorate were performed in the frequency range *f* = 120–10^5^ Hz and temperature interval 100–460 K with LCR-meter MIT 9216A (Protek Instrument Co., Ltd., Gyeoriggi-do, Republic of Korea), using the LabView software package (Version 2011, NIST, Gaithersburg, MD, USA). Silver glue was used for preparing the electrodes on the natural faces of the crystals. During thermal cycling, capacitance *C* and dielectric loss tangent tg*δ* were measured with a slow temperature change at a rate of 1–2 K/min. Cycles of computer-controlled measurements of *C* and tg*δ* at frequencies of *f* = 120 Hz, 1 kHz, 10 kHz and 100 kHz were repeated every 5 s.

## 3. Results

### 3.1. Single Crystal XRD Analysis

Single crystal XRD analysis shows that the MBI-perchlorate samples 1, 2 and 3 crystallize in monoclinic centrosymmetric lattice with space group *P*2_1_/*n* (14). Crystal data and structure refinement details of Sample 1 are summarized in Table 1. For Samples 2 and 3, crystal data and selected details of structure refinement are shown in Table S5 of the Supplementary Materials. Crystallographic information files (CIFs) for Samples 1, 2 and 3 were deposited at Cambridge Crystallographic Data Centre (2236941–2236943) and can be obtained free of charge via www.ccdc.cam.ac.uk/data_request/cif (accessed on 23 November 2022). In addition, CIFs for all samples are linked to Supplementary Materials. Coordinates of atoms and their equivalent isotropic/isotropic temperature factors *U*_eq_/*U*_iso_, atomic displacement parameters *U*^ij^, bond lengths and bond angles according to the results of the single crystal refinement of Sample 1 are presented in Tables S1–S4 of the Supplementary Materials (Section S.II).

**Figure 2:**
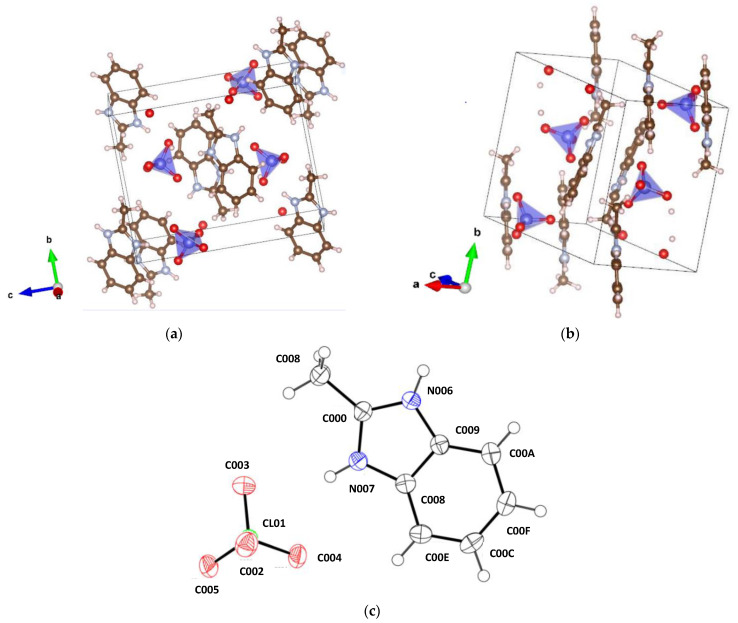
Images of MBI-perchlorate crystal structure (**a**,**b**). Perchlorate acid tetrahedra are marked by blue, red circles—O, brown circles—C, blue circles—N, open circles—H. (**c**) Perchloric acid and MBI molecule in MBI-perchlorate structure with atoms represented by thermal ellipsoids by means of program ORTEP [56] using the data presented in Tables S1–S4, correspondingly.

The unit cell contains four (C_8_H_8_N_2_) (HClO_4_) formula units (*Z* = 4 in Table 1). Crystal structure of MBI-perchlorate and images of HClO_4_ and MBI molecules with atoms represented by thermal ellipsoids are shown in Figure 2.

In the MBI-perchlorate structure, the flat MBI molecules (*C_s_*(*m*) symmetry) are arranged in antiparallel pairs. The ClO_4_ tetrahedra are located near the nitrogen atom in each MBI molecule. The planes of flat MBI molecules are oriented perpendicularly to (101) crystal plane and the planes of the MBI molecules are rotated in pairs at an angle of +24.3360(9)° or −24.336(9)° from the plane ($\bar{1}$01) of the crystal structure (Figure 2b). Since benzimidazoles are amphoteric, the interaction of MBI molecules with acids can be accompanied by protonation when a proton from HClO_4_ is transferred to one of the nitrogen ions of the MBI molecule. As a result, the (MBI + H)^+^ cation and the (ClO_4_)^–^ anion appear, and one double bond in the imidazole ring disappears. In particular, such a situation was observed in MBI-phosphite and MBI-phosphates [21].

Table 2 presents a comparison of the bond lengths and angles in the MBI molecule in crystals with (ImH_2_)SO_4_∙2H_2_O, MBI-phosphate-2, (ImH_2_)H_2_PO_4_)) and without protonation (MBI and Im), as well as in MBI-perchlorate (Sample 1). The main differences between protonated and non-protonated crystals are observed in N007-C00D-N006, C00D-N007-C008 and C00D-N006-C009 bond angles. In protonated MBI molecules, the bond angle N007-C00D-N006 is much smaller than in non-protonated ones. On the contrary, the angles C00D-N007-C008 and C00D-N006-C009 in protonated MBI molecules are larger. This situation is also observed for MBI-perchlorate (Table 2). Thus, we can conclude that the crystal structure of MBI-perchlorate is formed by (MBI + H)^+^ cations and (ClO_4_)^–^ anions packed into a lattice with four formula units.

Detailed comparison of interatomic distances and angles for samples 1, 2 and 3 is presented in Supplementary Materials (Tables S6 and S7, Section S.III). In all samples the bond lengths and bond angles of MBI molecular are very close.

Considerable differences are observed for structural parameters of perchlorate ClO_4_ tetrahedra. The ClO_4_ tetrahedra are most strongly distorted in sample 1. In large samples 2 and 3, they are closer to the shape of a regular tetrahedron. The unit cell volume in samples 2 and 3 is slightly bigger than in sample 1, which is mainly due to the larger values of parameters *a* and *b* and decrease in monoclinic angle *ß*. These differences may be caused by different concentration of impurities in samples, which can be introduced in voids of crystal structure during growth [32,37]. Apparently, these impurities are formed as a result of the decomposition of perchloric acid over time.

### 3.2. Powder XRD Analysis

The results of LPA by WHP and SSP methods showed the absence of microstrains in the crystallites and a rather large crystallite size of ~100 nm (Section S.I, Figure S1). LB-fitting of the MBI-perchlorate powder showed a fairly good quality (the agreement weight profile factor *R*_wp_ = 10.83%, Section S.I, Figure S2), which indicates that the powder is single-phase.

**Figure 3:**
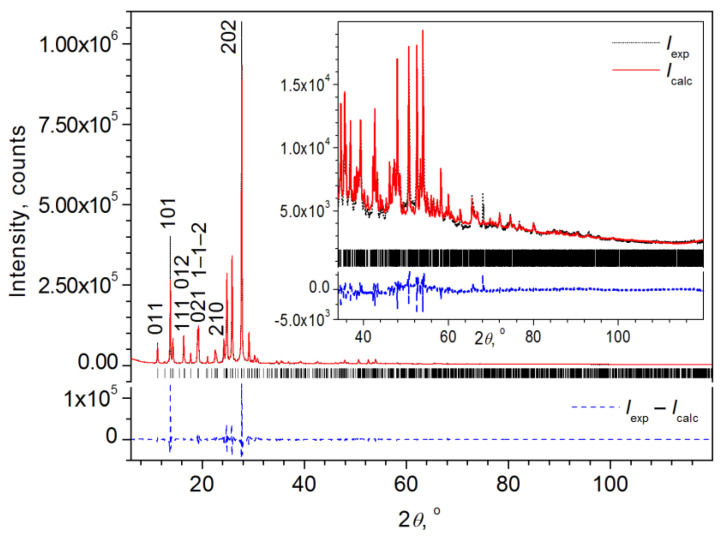
Final graphical results of Rietveld fitting of the MBI-perchlorate (space group *P*2_1_/*n* (14)) powder XRD pattern. The Bragg angle positions of all allowed reflections are shown by bar diagram. Miller indices *hkl* are indicated for some selected reflections (a list of *hkl* and squares of observed (*F*_hkl_obs_^2^) and calculated (*F*_hkl_calc_^2^) structure amplitudes as calculated by *TOPAS* is given in Supplementary Materials in a separate text-file with extention ‘fcf’).

The final results of the Rietveld refinement are shown in Figure 3 and in Table 3. Refined structure parameters (atomic coordinates and atomic temperature factors), as well as selected interatomic distances and angles are given in Tables S1, S3 and S4 of the Section S.I of the Supplementary Materials.

Thus, the Rietveld fitting of the XRD pattern and the refinement of the structure show a fairly good quality (the weight profile agreement factor *R*_wp_ is 12.67%, and the Bragg coefficient is 0.98%, Table 3). The volume *V*_cell_ of the unit cell of the MBI⋅HClO_4_ powder is 4.5% larger than that of a single crystal (compare Table 1 and Table 3) due to a noticeable increase in the unit cell parameters *a*, *c* and the angle *β* of monoclinicity while the parameter *b* varies slightly. Most likely, the volume of the unit cell of the powder is larger since its measurements took place at a temperature slightly higher than RT (313 K) while the single crystal at 100 K. If we turn to Figure 2a,b, which shows the structure, it can be seen that along the axes *a* and *c* are the largest voids in the structure while along the axis *b*, they are much smaller. This is probably the reason for a noticeable increase in parameters *a*, *c* and the angle *β* between them and a slight change in parameter *b* resulting in an increase of *V*_cell_ with an increase in the temperature *T*_meas_ of the XRD measurement of the powder pattern compared to single crystals.

As a result of the increase in the volume *V*_cell_ of the unit cell due to the higher measurement temperature *T*_meas_ in the XRD powder experiment compared to a single crystal one, the theoretical density *ρ*_calc_ of the MBI⋅HClO_4_ (Sample 1) is less in the case of XRD powder calculations than in the experiment with a single crystal (*cf.* 1.5539(1) g/cm^3^ and 1.6236(1) g/cm^3^ in Table 1 and Table 3, respectively). At the same time, the pycnometric density *ρ*_pyckn_ of a single crystal, measured at RT close to the XRD measurement temperature *T*_meas_ = 313 K of the powder, shows a value close to the theoretical value *ρ*_calc_ for the powder, but slightly less (cf. *ρ*_pyckn_ = 1.519(5) g/cm^3^ for single crystal Sample 2 and *ρ*_calc_ = 1.5539(1) g/cm^3^ according to powder XRD calculations for Sample 1). Thus, the compactness *C_m_* and porosity *P* of the MBI⋅HClO_4_ crystal are estimated to be 97.8(3)% and 2.246(3)%, respectively, which is comparable to the values of *C_m_* = 98.2% and *P*=1.8% in the inorganic perovskite-like La_0_._2_Bi_0_.2Ni_0_._5_Ti_0_._5_O_3_ [44].

Apparently, due to the higher temperatures *T*_meas_, the isotropic temperature factors *U*_iso_^overall^ of the atoms in the powder are higher than the equivalent isotropic temperature factors *U*_eq_ in the single crystal, which indicates a higher thermal oscillation of the atoms at a higher temperature (Table S1 of Supplementary Materials). As for the interatomic distances and angles (Tables S3 and S4 of Supplementary Materials), their average values in ClO_4_ and CH_3_ polyhedra and C_3_(NH)_2_ groups obtained for the powder are in fairly good agreement with the values determined on a single crystal. At the same time, for the powder, the individual values of the interatomic distances and angles show a noticeably greater spread around the average values than in the case of a single crystal. Apparently, this greater spread of individual values of interatomic distances and angles can be explained both as a result of the larger *T*_meas_ and by the fact that the single-crystal XRD method is much more precise for determining atomic coordinates, and hence the distances and angles between atoms, than the powder analogue.

### 3.3. Raman Scattering and FTIR Absorption Spectroscopy

**Figure 4:**
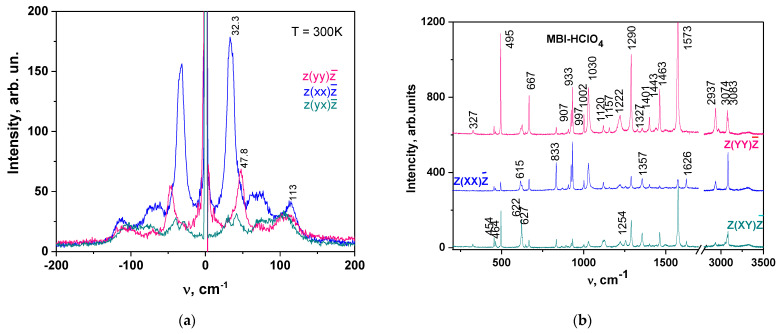
Raman spectra of MBI-perchlorate crystal for different experimental geometries. (**a**) Low energy region (−200–200 cm^−1^) with Stokes and anti-Stokes components, (**b**) Spectral region 100–3500 cm^−1^.

Raman scattering and FTIR absorption spectra observed in MBI-perchlorate (Sample 1) are similar to that in crystals whose structure involves MBI or perchloric acid molecules. Figure 4 presents polarized Raman spectra of MBI-perchlorate at RT for different experimental geometries after removing a background caused by relatively small luminescence at excitation at the wavelength *λ* = 532 nm. In spectral range *ν* = 100–3500 cm^−1^ the spectra consist of a large number of narrow lines caused in the region *ν* = 0–200 cm^−1^ by crystal lattice vibrations (Figure 4a) and at *ν* = 100–3500 cm^− 1^ intermolecular vibrations (Figure 4b).

**Figure 5:**
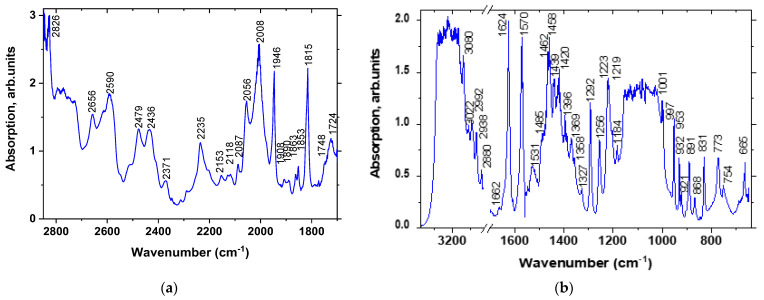
FTIR adsorption spectra of MBI-perchlorate crystals measured in transmission in thick (**a**) and thin (**b**) samples.

Measurements of FTIR absorption spectra were carried out using thick and thin samples (Sample 1). The thin sample (thickness *h*~0.1 mm) was measured in transmission mode, and the thick sample (*h*~0.5 mm) was measured in both transmission and specular reflection modes with subsequent Kramers-Kronig transformation. Transmission measurements on a thick sample made it possible to better determine the absorption peaks in the ranges of 1700–2850 and 3850–4800 cm^−1^. The absorption peaks in the ranges of 650–1700 cm^−1^ and 2850–3100 cm^−1^ are better seen on measurements of a thin sample. Figure 5 shows FTIR adsorption spectra of MBI-perchlorate crystals measured in transmission mode.

Raman shifts *ν* and wave numbers for FTIR absorption lines observed in MBI-perchlorate and their interpretation based on comparison with known spectra of MBI crystal [32,60] molecule and ClO_4_^−^ tetrahedron [56]) are presented in Table 4. Most of these lines (Figure 4 and Figure 5 and Table 4) correspond well to different types of MBI molecule vibrations.

It is important to note that according to XRD analysis, the MBI molecules in MBI- perchlorate are protonated, and both nitrogen atoms in the cation (MBI + H)^+^ form a valence bond with hydrogen atoms. This leads to disappearance of the double carbon–nitrogen valence bonds. There is no protonation in MBI single crystals, and a strong line at 1545 cm^−1^ is observed in the Raman spectrum caused by the stretching vibration of the carbon–nitrogen double valence bond mode in the imidazole ring [32]. In contrast, this line is practically absent in the spectrum of MBI⋅HlO_4_ (Table 4), which confirms the results of XRD analysis.

The free ClO_4_^−^-ion, having *T_d_* symmetry, has four main vibrations: a non-degenerate symmetric valence mode n1(A1), a doubly degenerate bending (deformation) mode n2(E), a three-fold degenerate asymmetric stretching mode n3(F2) and a three-fold degenerate asymmetric bending mode n4(F2). In the crystal, the ClO_4_ tetrahedron occupies a site of lower positional symmetry *C*1 and is strongly distorted (see Supplementary Materials Tables S6 and S7, Section S.III). As a result of low positional symmetry, the degeneracy of the n2, n3 and n4 modes can be lifted [61]. The nondegenerate valence mode n1 appears as a single band at 933 cm^−1^; the n2 mode appears at 464 cm^−1^ and 454 cm^−1^; the n3 mode appears as weak at 1128 cm^−1^, 925 cm^−1^ and 907 cm^−1^ and the n4 mode appears at 618 cm^−1^, 622 cm^−1^ and 647 cm^−1^.

To obtain the symmetry of the observed Raman lines, a study of angle-resolved Raman spectra was performed. To do this, the Raman spectra were recorded for Z(XX)$\;\bar{Z}$ and Z(XY)$\;\bar{Z}$ backscattering geometries, where Z is normal to the crystal plane (001) for both parallel (PP) and crossed (CP) orientation of polarizations with rotation of polarization around the normal to the surface with a step of 10° or 20°.

The MBI-perchlorate is characterized by monoclinic syngony (space group *P*2_1_/*n* (14)), and its Raman spectra show pronounced polarization dependence. For *P*2_1_*/n* (14) crystal symmetry, there are two irreducible representations, A_g_ and B_g_ for Raman active modes. In a widely adopted model [62,63,64,65] for arbitrary azimuth *θ* of sample the intensity of a polarized Raman signal is proportional to |**e_i_**·$\;\overset{↔}{R}\;$·**e_s_**|^2^, where **e**_i_ and **e**_s_ are the polarizations of the incident and scattered photons, respectively, and $\;\overset{↔}{R}\;$ is the complex Raman tensor for a given mode. For the PP case **e**_i_ = **e**_s_ = (cos*θ*, 0, sin*θ*) and for the CP one **e**_s_ = (–sin*θ*, 0, cos*θ*). The complex Raman tensors of the Raman active modes in the backscattering geometry are [63,64,65,66].

(4) $${\overset{↔}{R}}_{A_{g}}=\left[\begin{array}{ccc} \left|a\right|e^{i\varphi_{a}} & \left|d\right|e^{i\varphi_{d}} & 0\\ \left|d\right|e^{i\varphi_{d}} & \left|b\right|e^{i\varphi_{b}} & 0\\ 0 & 0 & \left|c\right|e^{i\varphi_{c}} \end{array}\right]$$

(5) $${\overset{↔}{R}}_{B_{g}}=\left[\begin{array}{ccc} 0 & 0 & \left|e\right|e^{i\varphi_{e}}\\ 0 & 0 & \left|f\right|e^{i\varphi_{f}}\\ \left|e\right|e^{i\varphi_{e}} & \left|f\right|e^{i\varphi_{f}} & 0 \end{array}\right]$$

where |*a*|, |*b*|, |*c*|, |*d*|, |*f*| are modulus of components of Raman tensors and *φ*_i_—corresponding phases. Expressions for angle variation of Raman intensity of lines of A_g_ and B_g_ symmetry are presented in Table 5.

In frames of this model for the PP case, the angle dependences of Raman intensity for Ag modes in polar coordinates reveal 180°-symmetry and is a sum of a circle and two pairs of petals oriented along 0 and 90°. The sizes of a circle and each pair of petals depend on |a|, |c| and (*φ*_c_ − *φ*_a_) parameters. For the CP case, angle variations reveal 90°-symmetry and are a sum of two pairs of petals of the same size oriented along 45° and 135°. Analogous shapes of angle dependence have B_g_ modes for the PP case. For the CP case, the petals are rotated on 45°.

**Figure 6:**
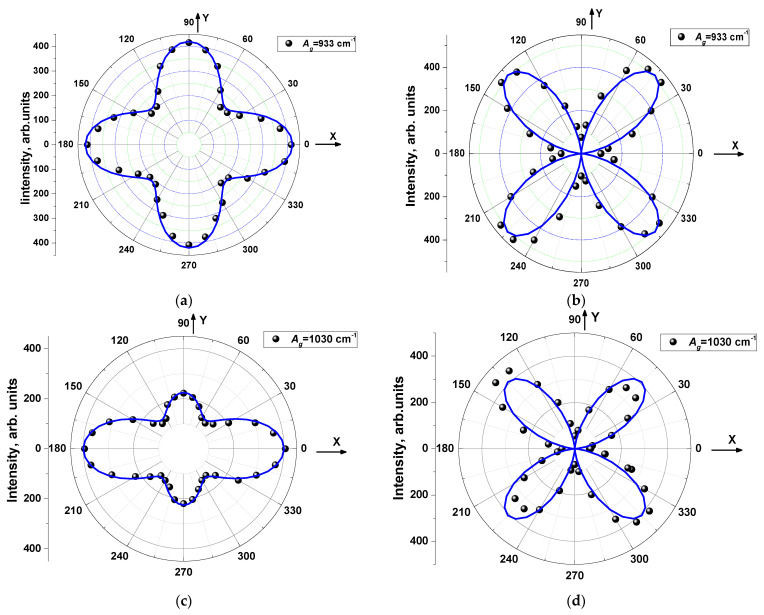

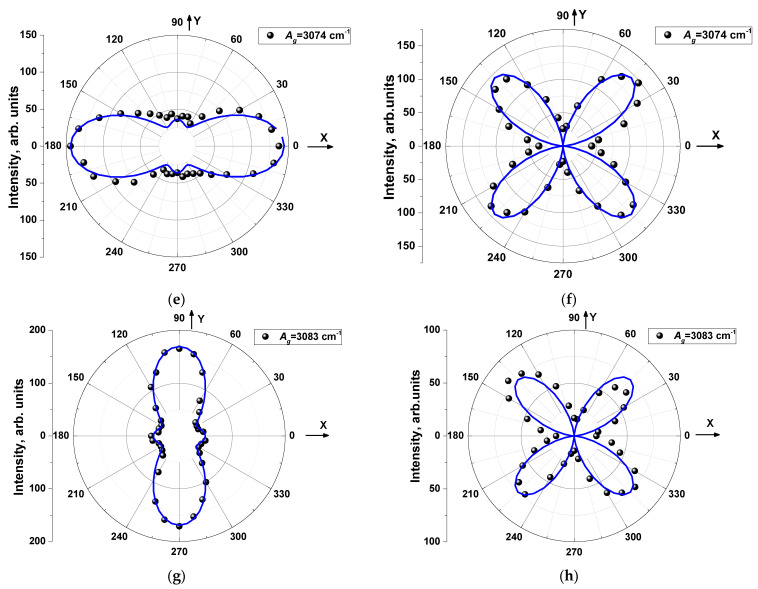
Angle variations of Raman intensity measured in PP (**a**,**c**,**e**,**g**) and CP (**b**,**d**,**f**,**h**) geometry for lines of Ag symmetry. Blue lines correspond to calculations using expressions (Table 5). (**a**,**b**) line *ν* = 933 cm^−1^, (**c**,**d**) line *ν* = 1030 cm^−1^, (**e**,**f**) line *ν* = 3074 cm^−1^, (**g**,**h**) line *ν* = 3083 cm^−1^.

**Figure 7:**
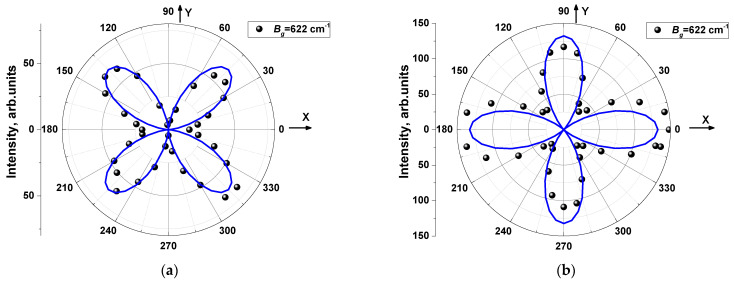
Angle variations of Raman intensity measured in PP (**a**) and CP (**b**) geometry for line *ν* = 622 cm^−1^ of B_g_ symmetry.

Most of experimental angular dependences of polarized Raman lines correspond well to predictions of the model [63,64,65,66], which makes it possible to get undoubtedly the symmetries of modes (Table 4). Examples of experimental angular dependence for A_g_ and B_g_ modes are shown in Figure 6 and Figure 7.

It should be noted that in the case of bulk transparent birefringent crystals, the simple model considered above, can only be applied to the orientation of the incident light **e_i_** parallel to one of the main directions of the section of the indicatrix by the reflecting plane of crystal. Only in this case, the polarization of the exciting and emitted light will not change over the thickness of the crystal. For other directions of polarization, the formulas of a simple model may inaccurately describe the angular dependences. Nevertheless, for a number of lines of Raman spectrum shown in Figure 4, this approach makes it possible to describe angular variations of the intensity of Raman lines. Using the expressions given in Table 5, we performed the fitting of the experimental angular dependences, and the results for some Raman lines are presented in Figure 6. From the fitting, we can conclude that for the A_g_ modes associated with vibrations of the MBI molecule, the values of the parameters |*a*| and |*c*| differ significantly. On the contrary, for A_g_ and B_g_ modes caused by vibrations in ClO_4_ tetrahedra, the values of these parameters are almost equal. This can be explained by the anisotropic nature of the vibrations (AI) of flat MBI molecules in the crystal structure of MBI-perchlorate as compared to the practically isotropic vibrations (I) of ClO_4_ tetrahedra.

It should be noted that the description of the angular dependences cannot be performed with good accuracy for all observed lines since some lines exhibit not 90°- but 180°-symmetry in the CP configuration. The reason may be related to the limitations of the simple model used and a more detailed approach should be applied [61]. However, the orientations of the principal axes (0°, 90° or 45°, 135°) on the angular dependences in both PP and CP configurations are in good agreement with the predictions of the simple model.

### 3.4. UV-Vis Absorption

**Figure 8:**
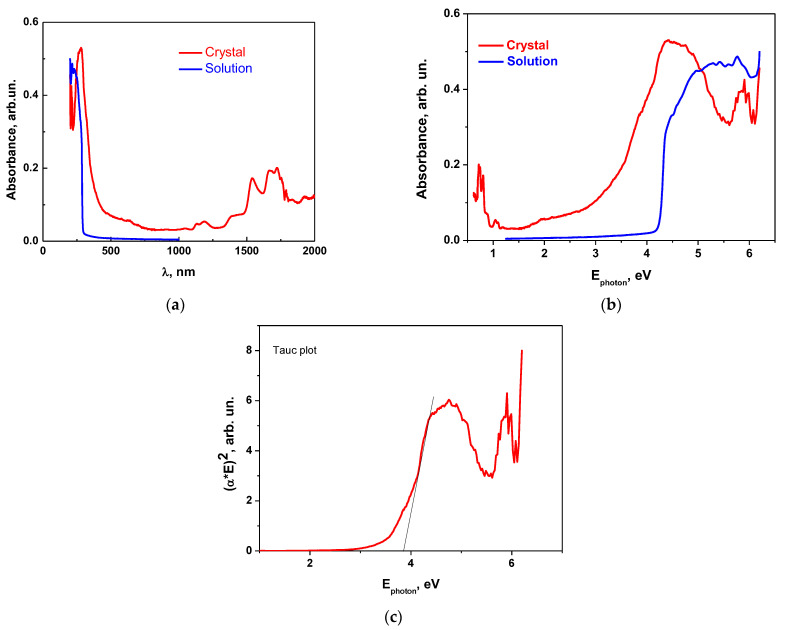
Absorbance spectra of MBI⋅HClO_4_ single crystal (Sample 1) and aqueous solution of MBI⋅HClO_4_ crystals (**a**,**b**). Tauc plot for direct allowed electronic transitions in MBI⋅HClO_4_ crystal (Sample 1) (**c**).

The absorbance (UV-Vis) spectra of MBI-perchlorate single crystal (Sample 1) and its aqueous solution are in general similar, although there are certain differences (Figure 8).

The absorbance (UV-Vis) spectra of MBI-perchlorate single crystal (Sample 1) and its aqueous solution are in general similar, although there are certain differences (Figure 8). Even in transparent colorless single crystals, an absorption edge is blurred as compared to that in the solution. An estimate of the optical band gap *E*_g_ based on the analysis of the Tauc plot for allowed direct electronic transitions in MBI⋅HClO_4_ (Figure 8c) yields the value *E*_g_ ≅ 3.9 eV, which is remarkably less than that in imidazole perchlorate films (*E*_g_ ≅ 5.4 eV), in which methyl groups are absent [26]. The value of *E*_g_ = 3.9 eV in the single crystal is markedly less than the width of the optical band gap of the solution (*E*_g_ = 4.2 eV, see Figure 8b), which can be related to a stronger interaction of molecules in the crystal.

The brown color of the large bulk crystal shown in Figure 1d (Sample 3) compared to the almost colorless crystal shown in Figure 1c (Sample 1) indicates the difference in impurity concentrations in the samples. The presence of impurities in the brown bulk crystal shown in Figure 1d is also confirmed by the photoluminescence spectra of the crystal (see below).

### 3.5. Photoluminescence

**Figure 9:**
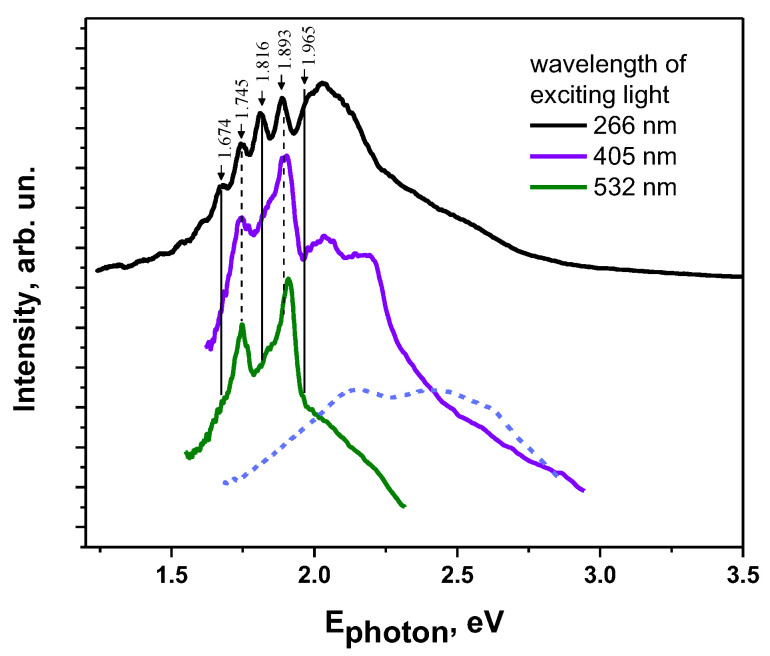
Photoluminescence spectra of MBI-perchlorate crystals (Sample 3) under photoexcitation with *λ*_ex_ = 266 nm, 405 nm, and 532 nm. The dotted line represents the emission spectrum of aqueous solution of brown MBI-perchlorate crystal under 405 nm excitation. The spectra are shifted along the vertical axis for the convenience of readers.

Figure 9 presents the photoluminescence (PL) spectra of MBI-perchlorate crystals upon excitation with light of different wavelengths. Obviously, the emission spectra explicitly depend on the excitation wavelength. The brightest emission was observed in our experiments with laser excitation with *λ_e_*_x_ = 266 nm (*hν*_ex_ = *E*_photon_ = 4.66 eV), corresponding to the region of fundamental absorption of the crystals. The PL spectra of samples 2 and 3 obtained under the UV excitation are almost identical and represent a broad weakly structured band with two maxima at 1.82 and 2.03 eV (Figure 9). The shape of the spectra suggests that the broad band is a superposition of several narrower overlapping bands (including the bands at 1.82 and 2.03 eV).

As can be seen from Figure 9, an increase in the excitation wavelength leads to a dramatic decrease in the intensity of the bands at 1.82 and 2.03 eV while two new relatively narrow emission bands with maxima at 1.745 and 1.905 eV flare up in the spectrum. Upon 405 nm excitation, an emission band at 2.18 eV also appears, which, in turn, disappears upon 532 nm excitation. Note that the relatively narrow bands (“lines”) observed in the emission spectra of colored crystals (*E*_photon_ = 1.90 eV and 1.75 eV) are not observed in the spectra of their aqueous solutions (Figure 9). Thus, such a “line” structure of the emission spectrum is a distinctive property of MBI-perchlorate crystals.

The pronounced effect of the excitation wavelength on the emission spectrum of the crystals under study can be associated with two factors. (i) Light absorption coefficients in the fundamental absorption region of crystals (*E*_photon_ > *E*_g_) and in their transparency region (*E*_photon_ < *E*_g_) are significantly different (Figure 8). As a result, under the conditions of UV excitation (*λ_e_*_x_ = 266 nm), the near-surface region of the crystals is predominantly excited, whereas when the crystals are excited by light with *λ_e_*_x_ = 405 nm and *λ_e_*_x_ = 532 nm, their volume is excited. However, the concentration and types of impurities and lattice defects can be quite different on the surface and in the bulk of the crystal, which can manifest itself in the difference of the emission spectra of its near-surface region and its bulk. (ii) When crystals are excited by light with *E*_photon_ > *E*_g_, the widest spectrum of excited electronic states (exciton and defect states) is involved (to a greater or lesser extent) in the process of radiative recombination. On the other hand, when crystals are excited by laser photons with *E*_photon_ < *E*_g_ (in the crystal transparency region), only defects with their optical absorption bands at *E* ≈ *E*_photon_ are selectively excited by the laser radiation, which generally narrows the emission spectrum.

The important role of impurity centers and lattice defects in the formation of the PL of the crystals under study is also confirmed by the following. When excited by light with *λ_e_*_x_ = 532 nm (*E*_photon_ = 2.33 eV), the intensity of the PL spectra of colorless crystals (sample 1) is very low (even lower than the intensity of the Raman lines), which clearly distinguishes them from the spectra of colored crystals (Figure 9). The high efficiency of photoexcitation of colored crystals by light with *λ_e_*_x_ = 532 nm indicates the presence of impurity/defect levels in the band gap of the crystals, providing effective absorption and emission of light with *λ* > 532 nm. The presence of this impurity absorption in the visible region of the spectrum determines the noticeable brown color of the crystals and indicates an increased concentration of impurities in the colored crystals. On the other hand, the change in the structure of the PL spectrum upon excitation with light with wavelengths of 405 or 532 nm compared to UV excitation demonstrates that the population of the emissive states of impurity centers depends significantly on the excitation wavelength.

### 3.6. Thermogravimetry-Differential Scanning Calorimetry (TG–DSC)

**Figure 10:**
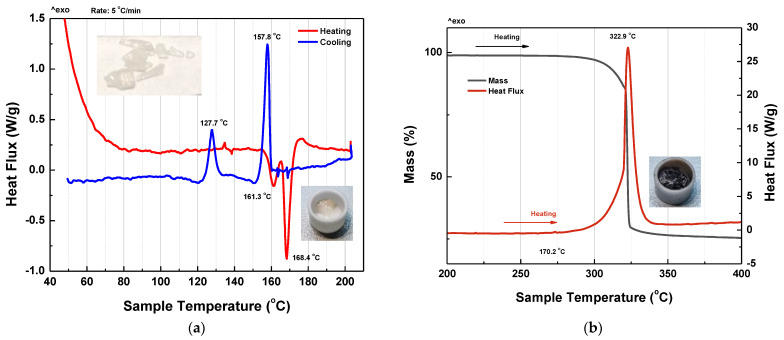
TG-DSC curves for heating and cooling at a rate of 5°/min of MBI-perchlorate crystals Sample 1 (**a**) DSC curves for heating up to 200 °C. Upper and Lower insets show images of sample before and after heating, respectively. (**b**) TG-DSC curves for heating from 200 °C up to 400 °C. The inset shows the image of sample after heating.

The TG and DSC curves of MBI-perchlorate crystals were measured in air. Figure 10 shows DSC curves for heating and cooling at a temperature change rate of 5°/min. Photographs of the sample before and after heating to 200 °C are shown in the top and bottom insets in Figure 10a.

The DSC curves clearly show two first-order phase transitions with different temperature hysteresis. The low-temperature phase transition occurs at *T*_c2_^h^ = 161.3 °C and *T_c2_^c^* = 127.7 °C (Δ*T*_c2_^hc^ = 33.6 °C) for heating and cooling, respectively. The high-temperature phase transition during heating occurs at *T*_c1_^h^ = 168.4 °C and at *T*_c1_^c^ = 157.8 °C during cooling (Δ*T*_c1_^hc^ = 10.6 °C). Since the samples lost their crystalline shape after heating up to 200 °C (see top and bottom insets in Figure 10a), we conclude that the melting point is the high-temperature transition. Above melting point, a liquid phase (LqPh) is realized.

A further increase in temperature up to 400 °C is accompanied by a sharp decrease in the mass of the sample at 322.9 °C (Figure 10b). This temperature can be considered as the decomposition temperature *T*_d_ of the sample. As a result of heating the sample up to 400 °C, a black residue remains, apparently carbon (see inset in Figure 10b). Thus, the TG-DSC experiments show that during melting, the samples retain their molecular composition. In the temperature range between 161.3 °C and 168.4 °C during heating and between 157.8 °C and 127.7 °C during cooling, the sample is in an intermediate solid phase (IPh), different from the phase at lower temperatures (LPh).

An analysis of the anomalies in the DSC curves at *T*_c1_^h^ and *T*_c2_^h^ gives the change in enthalpy Δ*H* = 57 Jg^−1^ and 28.6 Jg^−1^ and entropy Δ*S* = 30.7 J·mol^−1^·K^−1^ and 16.6 J·mol^−1^·K^−1^ *(*Δ*S =* Δ*H(M/T)*, where *M* is the molecular weight and *T* is the phase transition temperature), respectively. Large values of Δ*S* for both LPh → IPh and IPh → LqPh (liquid phase) phase transition indicate their order-disorder type. The value of Δ*S* = 30.7 J·mol^−1^·K^−1^ at the IPh → LqPh transition is comparable to that in benzene C_6_H_6_ Δ*S* = 38.0 J·mol^−1^·K^−1^ (*T*_m_ = 279.15 K).

The melting point of MBI *T*_m_ ~ 175–177 °C is somewhat higher than *T*_m_ = 168.4 °C in MBI-perchlorate. Comparing the melting temperature of MBI-perchlorate with other semi-organic perchlorates, some of which obey ferroelectric properties, it is seen that its *T*_m_ is much lower than that of imidazolium perchlorate [26], guanidinium perchlorate, [C(NH_2_)_3_]^+^ClO_4_^−^ [27], [2,4,6-trimethylpyridinium][ClO_4_], [(CH_3_)_3_C_5_H_2_NH][ClO_4_] [29], N,N’-diphenylguanidinium perchlorate [30] and so on, in which the melting temperature *T*_m_ is ~500 K (227 °C).

The irreversible transition at *T*_d_ = 322.9 °C is accompanied by a strong decrease in the mass of the sample up to 25%. Since the molar mass of C (carbon) in MBI⋅HClO_4_ is about 40%, this decrease corresponds to the volatilization of H, Cl, N, O ions and part of C ions. Only the carbon shown in the inset of Figure 10b remains. Obviously, this transition is not a simple evaporation, but it is accompanied by the decomposition of MBI molecules. For this reason, the enthalpy of transition is high, Δ*H* ~ 1500 J∙g^−1^.

The large difference between the decomposition temperature *T*_d_ and the melting point of MBI-perchlorate indicates that the properties of this material can be close to those of ionic liquids (ILs) [67,68] despite the fact that, by definition, the maximum temperature of ILs should be up to 100 °C.

### 3.7. Dielectric Properties

**Figure 11:**
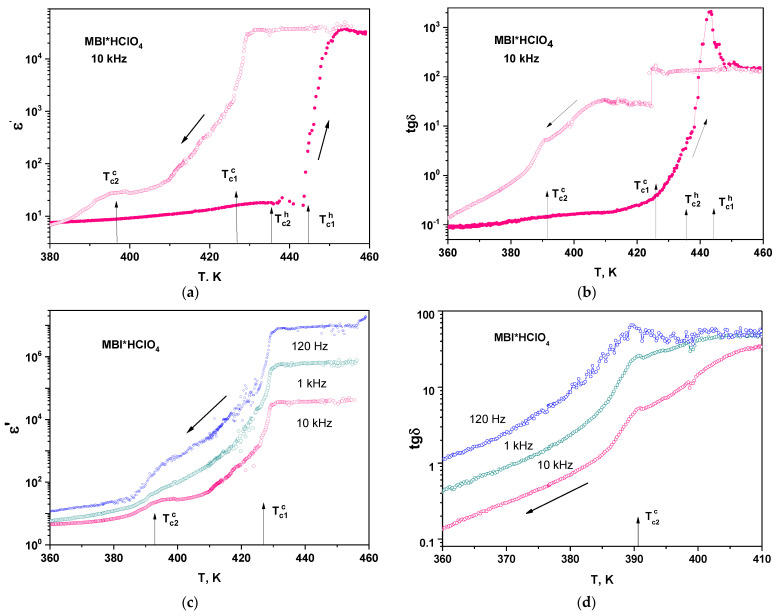
Temperature dependence of dielectric constant *ε*′ (**a**) and dielectric losses tg*δ* (**b**) in semi-logarithmic scales in MBI⋅HClO_4_ crystal during the heating-cooling cycle at frequency of 10 kHz. Temperature dependence of dielectric constant (**c**) and tg*δ* (**d**) for cooling at frequencies *f* of 120 Hz, 1 kHz, and 10 kHz.

Figure 11a,b shows the temperature dependence of dielectric constant *ε*’ and dielectric losses tg*δ* in MBI-perchlorate crystal during the heating-cooling cycle. It can be seen that when heated, the permittivity (*ε*′~8 and tg*δ*~0.1 at 380 K) slowly increase to temperature *T* ≅ 440 K (167 °C), after which there is a strong jump-wise increase by three orders of magnitude up to *ε*′~3 × 10^4^ and tg*δ*~100. In according with TG-DSC curves (Figure 10), this increase can be attributed to the melting of MBI-perchlorate. High values of dielectric constant *ε*′ at this phase transition may arise due to the effect of the ionic liquid (IL) [62], which appears during melting of MBI-perchlorate crystals at *T*_c1_^h^.

Since, as the DSC data show, the temperatures *T*_c2_^h^ and *T*_c1_^h^ of LPh → IPh and IPh → LqPh phase transitions are quite close against the background of a strong increase in *ε*′ and tg*δ*, it is not possible to identify the phase transition point *T*_c2_^h^. The phase transition temperature *T*_c1_^h^ obtained from dielectric measurements, shown in Figure 11a,b, is in good agreement with the DSC data, shown in Figure 10. The phase transition temperature *T*_c2_^h^ shown by arrows in Figure 11a,b was taken from DSC experiments.

Upon cooling, the high values of *ε*′ and tg*δ* in LqPh do not change up to *T*_c1_^c^*,* where a first-order phase transition LqPh → IPh occurs. At the temperature *T*_c1_^c^, there is a strong abrupt decrease in *ε*′ and tg*δ* by about an order of magnitude. Below *T* = 420 K and up to the phase transition temperature *T_c2_*^c^, a slow decrease and some stabilization of *ε*′ and tg*δ* is observed.

Figure 11c shows temperature dependence of dielectric constant *ε*′ at cooling at frequencies 120 Hz, 1 kHz and 10 kHz.

A strong frequency dispersion of *ε*′ in LqPh (*T > T*_c1_^c^) and in IPh (*T*_c2_^c^ < *T* < *T*_c1_^c^) is observed. The dispersion decreases below *T*_c2_^c^. In this temperature region, the temperature decrease is accompanied by a faster decrease in *ε*′ and tg*δ* than in the region *T > T*_c2_^c^ (Figure 11c,d). Most clearly, the anomaly manifests itself in the temperature dependence of tg*δ* (Figure 11d). The anomaly does not shift with frequency, indicating a phase transition rather than some relaxation process. The absence of jump-like behavior in dielectric constant and losses may be caused by the appearance of the polycrystal state, which can be realized in a cooling cycle after the melting point.

**Figure 12:**
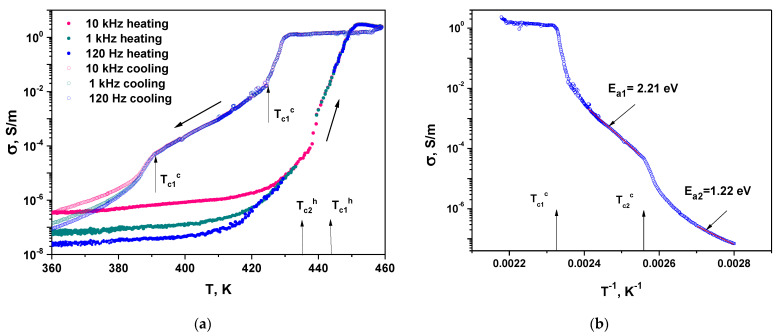
Temperature dependence of conductivity *σ* during heating–cooling cycle in semilogarithmic scale for different frequencies (**a**). Dependence of *σ* at *f* = 120 Hz on reversed temperature *T*^−1^ in semi-logarithmic scale (**b**).

Figure 12a shows the temperature dependence of the specific conductivity *σ* during the heating-cooling cycle at different frequencies on a semi-logarithmic scale. The conductivity was calculated from dielectric data as *σ* = *ω*·*ε*_0_·*ε*″ = *ω*·tg*δ*·*ε*_0_·*ε*′, where *ω = 2πf* is the frequency at which the dielectric data was measured. In contrast to dielectric constant, the conductivity in LqPh does not depend on both the temperature and frequency. Such behavior of conductivity was observed in ionic liquids based on imidazole at high temperatures in frequency interval of direct current (DC) conductivity [69]. The magnitude of conductivity in LqPh (*σ* = 2 S/m) is comparable with that in imidazolium ionic liquids (*σ* = 0.12 S/m–9 S/m).

In IPh, on cooling, the conductivity reveal strong temperature dependence, but the frequency dispersion is absent.

The frequency and temperature dependences of conductivity *σ* are described by the well-known expression [70,71].

*σ* = *σ*_DC_ + *A* · *ω^s^*, (6)

with temperature-dependent parameters *A* and *s*. The first term in Equation (6) is the DC conductivity, and the second term, describing the alternating current (AC) conductivity, is responsible for *σ* frequency dispersion. The absence of frequency dispersion in IPh and LqPh indicates the dominant contribution of DC conductivity. The conductivity frequency dispersion appears in LPh at *T* < *T*_c1_^h^ at heating and at *T* < *T*_c2_^c^ at cooling. Nevertheless, at cooling, the temperature dependences of *σ* at 120 Hz and 1 kHz almost coincide (Figure 12a), and we can use the temperature dependence of conductivity at 120 Hz to elucidate the activation energy both in IPh and LPh, using an Arrhenius law [70].

*σ*_DC_(*T*) = *σ*_0_*e*^−^*^E^*^a/*kT*^ (7)

Figure 12b shows the dependence of *σ* on the reversed temperature *T*^−1^ at 120 Hz in semi-logarithmic scale. Estimations show that the activation energy *E*_a_ in IPh is *E*_a1_= 2.21 eV and two times lower *E*_a2_= 1.22 eV in LPh. Note that the Arrhenius law is sometimes used for the product *σ*_DC_∙*T* [72]. However, in our case, taking into account the correction of the ln*T*-type for calculating *Ea* in the used temperature range turns out to be negligible.

Thus, the dielectric properties and conductivity of MBI⋅HClO_4_ crystals corresponds to results of TG-DSC measurements. Different phases observed in TG-DSC experiments (LPh, IPh and LqPh) reveal different the temperature and frequency behavior of dielectric constant, losses and conductivity. LqPh is characterized by frequency independent high values of dielectric constant and DC conductivity similar to ILs. Two solid phases exhibit temperature dependence of conductivity with different values of activation energy. In LPh, the activation energy *E_a_*_2_= 1.22 eV is very close to that for a protonic conductivity in ferroelectrics with hydrogen bonds [18,73,74]. The large values of the permittivity and activation energy in IPh may suggest the presence in this phase of atomic complexes, such as ClO_4_ tetrahedra or MBI molecules.

## 4. Conclusions

This study demonstrates that MBI-perchlorate crystals can be grown from an aqueous solution of crystalline 2-methylbebzimidazole (MBI or C_8_H_8_N_2_) and perchloric acid (HClO_4_) at RT. The obtained MBI-perchlorate crystals have a monoclinic centrosymmetric lattice, described by a space group *P*2_1_/*n* (14), with 4 (C_8_H_8_N_2_)(HClO_4_) formula units per the unit cell.

The presence of MBI and perchloric acid molecules in the composition of the crystals was confirmed by Raman spectroscopy and FTIR spectroscopy. A comparison of the spectra with those known from the literature makes it possible to attribute the observed lines to certain vibrations of the MBI molecule or the perchlorate tetrahedron. Angle-resolved polarized Raman spectroscopy enabled us to determine the symmetry (A_g_ or B_g_) of the observed Raman lines. An analysis of the XRD data and Raman spectra shows that the MBI molecules in the crystal structure of MBI-perchlorate are protonated and form anions while perchlorate acids stay as cations ClO_4_^−^.

With increasing temperature, MBI-perchlorate undergoes two first-order phase transitions at *T*_c2_^h^ = 161.3 °C and *T*_c1_^h^ = 168.4 °C with different temperature hysteresis (Δ*T*_c1_^hc^ = 10.6 °C and Δ*T*_c2_^hc^ = 33.6 °C). Higher temperature phase transition corresponds to the melting point. Thermal decomposition of the material on air occurs at *T* = 322.9 °C.

Absorption measurements in the UV-Vis region show that the optical band gap of MBI-perchlorate crystals is *E*_g_ ≅ 3.9 eV, which is slightly less than the optical band gap of an aqueous solution of MBI and HClO_4_ (*E*_g_ = 4.3 eV) and significantly less than that of imidazolium perchlorate (*E*_g_ ≅ 5.4 eV), in which there is no methyl group.

PL spectra of MBI-perchlorate crystals depend on the wavelength of exciting light. Going from UV excitation with *E_photon_* > *E_g_* to excitation by photons of lower energy with *E_photon_* < *E_g_* leads to a significant change in the shape of PL spectra, which is associated both with a difference in the concentration and types of luminescence centers on the surface and within crystals and with the selective excitation of certain luminescence centers under laser radiation with *E_photon_* < *E_g_*. It was also noted that, under excitation with *E_photon_* < *E_g_*, the PL of colored crystals is noticeably more intense than the PL of colorless samples, which seems to be associated with an increased concentration of luminescence centers in colored crystals. It can be assumed that the centers include chlorine oxides, which are the product of the partial decomposition of perchloric acid.

MBI⋅HClO_4_ crystals reveal remarkable dielectric anomalies at phase transitions. A particularly strong dielectric anomaly is observed at solid state–liquid transition, which is accompanied by strong jump-like increase by several orders of magnitude in dielectric constant and dielectric losses caused most probably by appearance of ionic liquid. The appearance of such a large capacitance may be of interest for the development of controlled capacitances since the sample can be heated by various methods, including heating by light radiation. The melting point of MBI-perchlorate is significantly lower than in other salts of perchloric acid, while the decomposition temperature is approximately the same. Therefore, the temperature range ΔT ≈ 150 K in which an ionic liquid can exist in MBI-perchlorate is larger than in other perchlorates. It is worth noting that thin films of MBI-perchlorate prepared on a different substrate with the evaporation method reveal some unusual dielectric properties, but the results from their investigation need separate thorough investigation and will be published elsewhere.

## Figures and Tables

**Table 1:** Details of crystal data and structure refinement of MBI-perchlorate single crystal (Sample 1).

	MBI-Perchlorate
Chemical formula	C_8_H_9_ClN_2_O_4_
Formula weight, Da	232.62
Space group	*P*2_1_*/n* (14)
*a*, Å	7.6940(2)
*b*, Å	9.9774(2)
*c*, Å	12.4450(4)
*β*, *°*	94.674(3)
*V*_cell_, Å^3^	952.17(4)
*Z*	4
*ρ*_calc_, g/cm^3^	1.6236(1)
*F*(000)	480.0
*μ*, mm^−1^	3.582
Radiation (*λ*, Å)	Cu-*K_α_* (1.54184)
*Θ* max, °	70.391
*h*, *k*, *l* max	9, 10, 15
Reflections collected	5321
Independent reflections	1812
Data/restraints/parameters	1812/0/137
*GoF*	1.105
Final *R* indexes [Reflections *I* >= 2*σ*(I)]	*R*_1_ = 0.0499, *wR*_2_ = 0.1399
Final *R* indexes [Reflections all]	*R*_1_ = 0.0553, *wR*_2_ = 0.1477
Largest diff. peak/hole, *e*∙Å^−3^	0.64/−0.60
Temperature of measurements, *T*_meas_, K	100 ± 2

**Table 2:** Bond lengths and angles of MBI molecule in crystals with and without protonation.

Crystal	Bond, Å C00D-N007	Bond, Å C00D-N006	Angle, ° N007-C00D-N006	Angle, ° C00D-N007-C008	Angle, ° C00D-N006-C009
MBI [57]	1.339	1.335	112.7	106.1	106.3
Imidazole (Im) [31]	1.337(3)	1.316(2)	112.0(1)	107.3	105.3
(ImHz)SO_4_·2H_2_0 [58]	1.323(4)	1.333(4)	108.4(2)	108.7(2)	108.2(2)
MBI-phosphate-2 [21]	1.3299(13)	1.3347(13)	109.38(9)	109.19(8)	108.96(8)
(ImH_2_)H_2_PO_4_ [59]	1.320(4)	1.320(4)	108.7(3)	108.6(6)	108.6(6)
MBI⋅HClO_4_ (Sample 1)	1.330(3)	1.330(3)	108.6(2)	109.6(2)	109.8(2)

**Table 3:** MBI-perchlorate (*T*_meas_ = 313 ± 1 K). Monoclinic unit cell parameters (*a*, *b*, *c*, and *β*), unit cell volume *V*_cell_, mean crystallite size *D*, and reached agreement factors [55] obtained in Rietveld refinement and the factor *m*_e.s.d._ for correction of e.s.d.s of refined parameters.

*a*, Å *b*, Å	*c*, Å *β*, °	*V*_cell_, Å^3^ *D*, nm	*ρ*_calc_, g/cm^3^	*R*_w*p*_, % *R*_p_, %	*cR*_wp_, % ^a^ *cR*_p_, % ^a^	*R*_B_, % *m*_e.s.d._ ^a^
7.9123(1) 9.9617(5)	12.6787(3) 95.404(1)	994.89(6) 93(1)	1.5539(1)	12.67 9.29	18.57 16.59	0.98 4.127

^a^ Parameters calculated by program *RietESD* (see Ref. [45] and Supplementary Materials).

**Table 4:** Raman shifts and FTIR absorption lines (cm^−1^) observed in MBI-perchlorate. Last column shows interpretation of the FTIR lines according to MBI assignment [60]. Abbreviations: vs—very strong, s—strong, m—medium, w—weak, b—broad, *Γ*—out-of-plane bending, δ—in-plane bending, ν—stretching, M—methyl group.

Raman Z(YY)$\bar{Z}$	Raman Z(XX) $\bar{Z}$	Raman Z(XY) $\bar{Z}$	Symmetry Raman Modes	FTIR	Assignment
3358–3206b	-‘-	-‘-	A_g_ (3318)	3136–3373b	MBI: ν_NH_, ν_CH_, Mν_CH_
3083s 3074s	3083s 3047w	3083m 3074m	A_g_ (AI) A_g_ (AI)	3080m	MBI: ν_CH_
	3049vw	3049w		3022w	MBI: Mν_CH_
2971w		2972vw		2992w	MBI: Mν_CH_
2937s	2938w	2935w	A_g_	2938m	MBI: Mν_CH_
				2880m	
				2826w	
				2729–2792b	
				2656m	
				2590m	
				2479m	
				2436m	
				2371m	
				2235m	
				2153w	
				2118w	
				2087m	
				2056s	
				2008s	
				1946s	
				1908w	
				1890w	
				1863 m	
				1853m	
				1815m	
				1748sh	MBI: δ_NH_
				1724m	MBI: δ_NH_
				1662m	MBI: δ_NH_
1625vw	1626m	1626w	A_g_ (AI)	1624vs	MBI: ν_CC_ + ν_CN_
1573vs	1573m	1576s	A_g_ (AI)	1570vs	MBI: ν_CC_
1549vw				1531m	MBI: δ_HCC_ + ν_CC_
1505vw 1495vw	1503vw 1497vw	1501vw 1497vw		1485sh	MBI: Mδ_CH2_ MBI: Mδ_CH2_ + Γ_CCCN_ + δ_CCH_
1463s	1462w	1463m	A_g_ (AI)	1462s 1458vs	MBI: δ_CCH_ + ν_CC_
1443vw 1439vw		1444vw 1435vw		1439m	MBI: δ_CNH_ + ν_CN_ + ν_CC_ + _δCCH_
		1420vw	A_g_ (AI)	1420vs	MBI: Mδ_CH2_ + δ_CCH_
1401m	1400w	1401w	A_g_ (AI.)	1396m	MBI: Mδ_CH2_ + δ_CCH_
1356w	1357m	1356m	Ag (AI)	1369m 1358m	MBI: ν_CC_ + ν_CN_ + Mδ_CH2_
1327vw	1326vw	1327vw		1327m	MBI: δ_CCH_ + ν_CN_
1290vs	1290m	1290s	A_g_ (AI)	1292s	MBI: ν_CN_ + ν_CC_ + δ_CCH_
1255vw	1255w	1254m		1256s	MBI: δ_CCH_ + ν_CN_ + ν_CC_
1222m	1220w	1223w	A_g_ (AI)	1223s 1219s	MBI: δ_CNH_ +ν_CN_ + ν_CC_
1155w	1157w	1153vw	A_g_ (AI)	1184s	MBI: δ_CCH_
		1128w	B_g_ (I)		ν_as_(ClO_4_) v3(F2)
1120w	1120m	1118w	A_g_ (AI)	1153–1020b	MBI: δ_CCH_ + ν_CC_
1030s	1030s	1030m	A_g_ (AI)		MBI: ν_CC_ + ν_CN_
1002m 997m	1002m 999m	1002w 997w	A_g_ (AI) A_g_ (AI)	1001s 997s	MBI: ν_CC_+ δ_CCH_
958w	968vw			953s	MBI: *Γ*_HCCH_ + *Γ*_CCCH_
933s	933s	933m	A_g_ (I)	932s	ν_s_(ClO_4_) v1(A1)
924s	924s	925w	A_g_ (I)	921m	ν_as(_ClO_4_) v3(F2)
907w	907m	906vw	A_g_ (I)		ν_as_(ClO_4_) v3(F2)
893w	891vw	891vw		891s	MBI: δ_CCC_ + δ_CCH_
866w	870vw	865vw		868m	MBI: *Γ*_CCCH_ + *Γ*_HCCN_+ Γ_HCCH_ + *Γ*_CCCH_
833m	833s	834m	A_g_ (AI)	831s	MBI: ν_CC_
754s				773s 754m	MBI: *Γ*_CCCH_ + *Γ*_CCCC_ MBI: *Γ*_HCCH_ +*Γ*_CCCH_
667s	667m	667m	A_g_(AI)	665s	MBI: ν_CC_ + δ_CCC_ + δ_CCN_
658vwsh	658vw		A_g_ (AI)		MBI: *Γ*_CNCN_ + *Γ*_HCCN_ + *Γ*_CCCN_ + *Γ*_HNCN_
647w	647vw	647w	A_g_ (AI)		δ_as_(ClO_4_) V4(F1)
630m		627s	A_g_(AI)		MBI: δ_CCN_ + δ_CCC_ + ν_CC_
626m	624m	622s	B_g_ (I)		δ_as_(ClO_4_) V4(F1)
618m	615m	618sh	A_g_(AI)		δ_as_(ClO_4_) V4(F1)
495vs	494m	496s	A_g_		MBI: δ_CCC_ + ν_CC_ + ν_CN_
464w 454m	463m 454m	464m 455m	B_g_ A_g_		δ_s_(ClO_4_) V2(E) δ_s_(ClO_4_) V2(E)
434m	434w		A_g_		MBI: *Γ*_CCCC_ + *Γ*_CCCH_ + *Γ*_CCNH_ + *Γ*_CCNC_
327m	325vw	324w			MBI: *Γ*_CCCN_ + *Γ*_CCNH_
		279vw			MBI: δ_CCN_
161w		157vw			MBI: *Γ*_CCCN_ + *Γ*_HCCN_?
112s	113s	110sh	A_g_		Lattice mode
103s	101s	102s	B_g_		Lattice mode
		83w	B_g_		Lattice mode
73w	74w	74w			
69	69		A_g_		Lattice mode
	61				Lattice mode
47.7	47.8	41.6	A_g_		Lattice mode
	32.3s	30.5w			Lattice mode

**Table 5:** Expressions for angle dependence of Raman lines of A_g_ and B_g_ symmetry for parallel and crossed polarizers.

	A_g_	B_g_
Parallel polarizations (PP)	*I* ~{|a|cos^2^*θ* + |c| sin^2^*θ* · cos(*φ*_c_ − *φ*_a_)}^2^ + |c|^2^ sin^4^*θ* · sin^2^(*φ*_c_ − *φ*_a_)	|e|sin^2^2*θ*
Crossed polarizations (CP)	*I* ~1/4·[{|a|– |c| cos(*φ*_c_ − *φ*_a_)}^2^ + |c|^2^ sin^2^(*φ*_c_ − *φ*_a_)] sin^2^2*θ*	|e|cos^2^2*θ*

## Data Availability

The data presented in this study are available on request from the corresponding author.

## Supplementary Materials

The following supporting information can be downloaded at: https://www.mdpi.com/article/10.3390/ma16051994/s1, S.I Details of powder XRD experiment, XRD line profile analysis, and crystal structure refinement, Figure S1. (a) WHP and (b) SSP graphs for MBI-perchlorate, Figure S2. Final graphical results of LB fitting of the MBI-perchlorate (space group *P*2_1_/*n* (14)) powder XRD pattern, S.II Crystal structure parameters of MBI-perchlorate according to single crystal and powder X-ray diffraction data, Table S1. Results of refinement of the MBI-perchlorate (MBI⋅HClO_4_) structure, Table S2. Anisotropic atomic displacement parameters *U_ij_* for the MBI⋅HClO_4_ single crystal Sample 1, Table S3. Bond angles (°) in MBI-Perchlorate structure according to results of structure refinement using single crystal and powder XRD data (Table S1) for Sample 1. Temperature *T*_meas_ of data collection is indicated, Table S4. Bond lengths (Å) in MBI⋅HClO_4_ structure according to results of structure refinement using single crystal and powder XRD data (Table S1.1) for Sample 1, S.III. Comparison of crystal structure parameters in different samples of MBI⋅HClO_4_ single crystals, Figure S3. Optical images of (a) small transparent colorless plate Sample 1, (b) large Sample 2 with faint pink color, (c) thick bulk crystal (Sample 3) shown with distinct brown tint, Table S5. Unit cell parameters and volume and selected experimental and structure refinement details of different samples of MBI⋅HClO_4_ single crystals, Table S6. Selected bond lengths and angles of MBI molecule in different samples of MBI⋅HClO_4_ single crystals, Table S7. Selected bond lengths and angles of perchlorate tetrahedron ClO_4_ in different samples. S.IV. TG—DSC curves in MBI⋅HClO_4_. Figure S4. TG-DSC curves for heating and cooling of MBI-perchlorate crystals Sample 1 measured at a rate of 20°/min (a), 10°/min (b), and 5°/min (c). References [32,39,40,41,42,43,47,48,49,50,51,52,53,54,55] are cited in Supporting Materials.

## References

[1] Vijayakanth T., Liptrot D.J., Gazit E., Boomishankar R., Bowen C.R. (2022). Recent Advances in Organic and Organic–Inorganic Hybrid Materials for Piezoelectric Mechanical Energy Harvesting. Adv. Funct. Mater..

[2] Naber R.C.G., Asadi K., Blom P.W.M., de Leeuw D.M., de Boer B. (2010). Organic nonvolatile memory devices based on ferroelectricity. Adv. Mater..

[3] Park C., Lee K., Koo M., Park C. (2020). Soft Ferroelectrics Enabling High Performance Intelligent Photo Electronics. Adv. Mater..

[4] Lee Y.H., Kweon O.Y., Kim H., Yoo J.H., Hana S.G., Oh J.H. (2018). Recent advances in organic sensors for health self-monitoring systems. J. Mater. Chem. C.

[5] Sun Z., Luo J., Zhang S.H., Ji C.H., Zhou L., Li S.H., Deng F., Hong M. (2013). Solid-State Reversible Quadratic Nonlinear Optical Molecular Switch with an Exceptionally Large Contrast. Adv. Mater..

[6] Ghazaryan V.V., Zakharov B.A., Petrosyan A.M., Boldyreva E.V.L. (2015). Argininium phosphite—A new candidate for NLO materials. Acta Crystallogr..

[7] Haile S.M., Boysen D.A., Chisholm C.R.I., Merle R.B. (2001). Solid acids as fuel cell electrolytes. Nature.

[8] Blazcues-Kastro A., Garcia-Kabanes A., Carracosa M. (2018). Biological applications of ferroelectric materials. Appl. Phys. Rev..

[9] Horiuchi S., Kagawa F., Hatahara K., Kobayashi K., Kumai R., Murakami Y., Tokura Y. (2010). Above-room-temperature ferroelectricity in a single-component molecular crystal. Nature.

[10] Horiuchi S., Kobayashi K., Kumai R., Ishibashi S. (2017). Proton tautomerism for strong polarization switching. Nat. Commun..

[11] Horiuchi S., Kagawa F., Hatahara K., Kobayashi K., Kumai R., Murakami Y., Tokura Y. (2012). Above-room-temperature ferroelectricity and antiferroelectricity in benzimidazoles. Nat. Commun..

[12] Saripalli R.K., Swain D., Prasad S., Nhalil H., Bhat H.L., Guru Row T.N., Elizabeth S. (2017). Observation of ferroelectric phase and large spontaneous electric polarization in organic salt of diisopropylammonium iodide. J. Appl. Phys..

[13] Baran J., Bator G., Jakubas R., Sledz M. (1996). Dielectric dispersion and vibrational studies of a new ferroelectric, glycinium phosphite crystal. J. Phys. Condens. Mater..

[14] Matthias B.T., Miller C.E., Remeika J.P. (1956). Ferroelectricity of glycine sulfate. Phys. Rev..

[15] Albers J., Klopperpieper A., Rother H.J., Haussühl S. (1988). Ferroelectricity in betaine phosphite. Ferroelectrics.

[16] Banys J., Macutkevic J., Klimm C., Völkel G., Kajokas A., Brilingas A., Grigas J. (2004). Dielectric properties in the vicinity of the ferroelectric phase transition in a mixed crystal of deuterated betaine phosphate 0.03 betaine phosphite 0.97. Phys. Stat. Sol..

[17] Albers J., Klpperpieper A., Müser H.E., Rother H.J. (1984). Ferroelectric and antiferroelectric properties of deuterated betaine arsenate and betaine phosphate. Ferroelectrics.

[18] Balashova E.V., Krichevtsov B.B., Yurko E.I., Svinarev F.B., Pankova G.A. (2015). Dielectric properties of ferroelectric betaine phosphite crystals with a high degree of deuteration. Phys. Solid State.

[19] Averbuch-Pouchot M.T. (1993). Crystal structure of L-histidinium phosphite and a structure reinvestigation of the monoclinic form of L-histidine. Z. Kristallogr..

[20] Balashova E.V., Krichevtsov B.B., Popov S.N., Brunkov P.N., Pankova G.A., Zolotarev A.A. (2018). Elastic and Piezoelectric Parameters of the Crystals of Histidine Phosphite L-Hist H_3_PO_3_ Measured by the Method of Electromechanical Resonance. Tech. Phys. Lett..

[21] Balashova E.V., Svinarev F.B., Zolotarev A.A., Levin A.A., Brunkov P.N., Davydov V.Y., Smirnov A.N., Redkov A.V., Pankova G.A., Krichevtsov B.B. (2019). Crystal structure, Raman spectroscopy and dielectric properties of new semi-organic crystals based on 2-methylbenzimidazole. Crystals.

[22] Wayne W.C. (1973). Perchlorate salts, their uses and alternatives. J. Chem. Educ..

[23] Matthews L. (2011). Perchlorates: Production, Uses and Health Effects.

[24] Gault S., Jaworek M.W., Winter R., Cockel C.S. (2021). Perchlorate salts confer psychrophilic characteristics in α-chymotrypsin. Sci. Rep..

[25] Bishop L.J., Quinn R., Dyar M.D. (2014). Spectral and thermal properties of perchlorate salts and implications for Mars. Am. Mineral..

[26] Pająk Z., Czarnecki P., Szafrańska B., Małuszyńska H., Fojud Z. (2006). Ferroelectric ordering in imidazolium perchlorate. J. Chem. Phys..

[27] Szafrański M. (2011). Simple Guanidinium Salts Revisited: Room-Temperature Ferroelectricity in Hydrogen-Bonded Supramolecular Structures. J. Phys. Chem. B.

[28] Gao W., Chang L., Ma H., You L., Yin J., Liu J., Liu Z., Wang J., Yuan G. (2015). Flexible organic ferroelectric films with a large piezoelectric response. NPG Asia Mater..

[29] Wojtas M., Czupiński O., Tylczyński Z., Isakov D., Belsley M., Jakubas R. (2014). Optical nonlinearity and piezoelectricity in 2,4,6-trimethylpyridinium perchlorate. Chem. Phys..

[30] Kajamuhideen M.S., Sethuraman K., Ramasamy P. (2018). Crystal growth, physical properties and computational insights of semi-organic non-linear optical crystal diphenylguanidinium perchlorate grown by conventional solvent evaporation method. J. Cryst. Growth.

[31] Craven B.M., McMullan R.K., Bell J.D., Freeman H.C. (1977). The Crystal Structure of Imidazole by Neutron Diffraction at 20 °C and −150 °C. Acta Crystallogr..

[32] Balashova E., Levin A.A., Fokin A., Redkov A., Krichevtsov B. (2021). Structural Properties and Dielectric Hysteresis of Molecular Organic Ferroelectric Grown from Different Solvents. Crystals.

[33] Sheldrick G.M. (2008). A short history of SHELX. Acta Crystallocr. A.

[34] Dolomanov O.V., Bourhis L.J., Gildea R.J., Howard J.A.K., Puschmann H. (2009). OLEX2: A complete structure solution, refinement and analysis program. J. Appl. Crystallogr..

[35] Momma K., Izumi F. (2011). VESTA 3 for three-dimensional visualization of crystal, volumetric and morphology data. J. Appl. Crystallogr..

[36] Balashova E.V., Levin A.A., Davydov V.Y., Smirnov A.N., Starukhin A.N., Pavlov S.I., Krichevtsov B.B., Zolotarev A.A., Zhang H., Li F. (2022). Croconic acid doped triglycine sulfate single crystals: Crystal structure, UV VIS, FTIR, Raman, photoluminescence spectroscopy, and dielectric properties. Crystals.

[37] Balashova E., Levin A.A., Davydov V., Smirnov A., Starukhin A., Pavlov S., Krichevtsov B., Zolotarev A., Zhang H., Li F. (2022). Croconic Acid Doped Glycine Single Crystals: Growth, Crystal Structure, UV-Vis, FTIR, Raman and Photoluminescence Spectroscopy. Crystals.

[38] Diffrac (2019). Suite Eva.

[39] Maunders C., Etheridge J., Wright N., Whitfield H.J. (2005). Structure and microstructure of hexagonal Ba_3_Ti_2_RuO_9_ by electron diffraction and microscopy. Acta Crystallogr. B.

[40] Terlan B., Levin A.A., Börrnert F., Simon F., Oschatz M., Schmidt M., Cardoso-Gil R., Lorenz T., Baburin I.A., Joswig J.-O. (2015). Effect of Surface Properties on the Microstructure, Thermal, and Colloidal Stability of VB_2_ Nanoparticles. Chem. Mater..

[41] Terlan B., Levin A.A., Börrnert F., Zeisner J., Kataev V., Schmidt M., Eychmüller A. (2016). A Size-Dependent Analysis of the Structural, Surface, Colloidal, and Thermal Properties of Ti_1–x_B_2_ (x = 0.03–0.08) Nanoparticles. Eur. J. Inorg. Chem..

[42] Levin A.A. (2022). Program SizeCr for Calculation of the Microstructure Parameters from X-ray Diffraction Data. Preprint. https://www.researchgate.net/profile/Alexander-Levin-6/research.

[43] Langford J.I., Cernik R.J., Louer D. (1991). The Breadth and Shape of Instrumental Line Profiles in High-Resolution Powder Diffraction. J. Appl. Phys..

[44] Gharbi S., Dhahri R., Rasheed M., Dhahri E., Barille R., Rguiti M., Tozri A., Berber M.R. (2021). Effect of Bi substitution on nanostructural, morphologic, and electrical behavior of nanocrystalline La_1-x_Bi_x_Ni_0.5_Ti_0.5_O_3_ (x = 0 and x = 0.2) for the electrical devices. Mater. Sci. Eng. B.

[45] Le Bail A., Duroy H., Fourquet J.L. (1988). Ab-initio structure determination of LiSbWO6 by X-ray powder diffraction. Mat. Res. Bull..

[46] Rietveld H.M. (1967). Line profiles of neutron powder-diffraction peaks for structure Refinement. Acta Crystallogr..

[47] (2014). TOPAS.

[48] Cheary R.W., Coelho A.A. (1992). A fundamental parameters approach to X-ray line-profile fitting. J. Appl. Crystallogr..

[49] Balzar D., Snyder R.L., Fiala J., Bunge H.J. (1999). Voigt-function model in diffraction line-broadening analysis. Defect and Microstructure Analysis by Diffraction.

[50] Dollase W.A. (1986). Correction of Intensities for Preferred Orientation in Powder Diffractometry: Application of the March Model. J. Appl. Crystallogr..

[51] Järvinen M. (1993). Application of symmetrized harmonics expansion to correction of the preferred orientation effect. J. Appl. Crystallogr..

[52] Berger H. (1986). Study of the K alpha emission spectrum of copper. X-ray Spectrom..

[53] Bérar J.-F., Lelann P.J. (1991). ESD’s and Estimated Probable Error Obtained in Rietveld Refinements with Local Correlations. J. Appl. Crystallogr..

[54] Levin A.A. (2022). Program RietESD for Correction of Estimated Standard Deviations Obtained in Rietveld-Refinement Program. Preprint. https://www.researchgate.net/publication/359342753_Program_RietESD_for_correction_of_estimated_standard_deviations_obtained_in_Rietveld-refinement_programs.

[55] Hill R.J., Fischer R.X. (1990). Profile agreement indices in Rietveld and pattern-fitting analysis. J. Appl. Crystallogr..

[56] Farrugia L.J. (1997). ORTEP-3 for Windows—A version of ORTEP-III with a Graphical User Interface (GUI). J. Appl. Crystallogr..

[57] Obodovskaya A.E., Starikova Z.A., Belous S.N., Pokrovskaya I.E. (1991). Crystal and molecular structure of 2-methylbenzimidazole. J. Struct. Chem..

[58] Freeman H.C., Huq F., Rosalky J.M., Taylor I.F. (1975). The Crystal and Molecular Structure of Imidazolium Sulphate Dihydrate. Acta Cryst..

[59] Blessing H.R., McGandy E.L. (1972). Base stacking and hydrogen bonding in crystals of imidazolium dihydrogen orthophosphate. J. Amer. Chem. Soc..

[60] Güllüoglu M.T., Özduran M., Kurt M., Kalaichelvan S., Sundaraganesan N. (2010). Molecular structure and vibrational spectra of 2- and 5-methylbenzimidazole molecules by density functional theory. Spectrochim. Acta Part A.

[61] Antić-Jovanović A., Jeremic M., Lalic M., Long D.A. (1989). Raman Spectral Study of the Mg(C104)2-NaNCS-H20 System at Ambient and Elevated Temperatures. J. Raman Spectrosc..

[62] Sugai S., Shirotani I. (1985). Raman and infrared reflection spectroscopy in black phosphorus. Solid State Commun..

[63] Ribeiro-Soares J., Almeida R.M., Cançado L.G., Dresselhaus M.S., Jorio A. (2015). Group theory for structural analysis and lattice vibrations in phosphorene systems. Phys. Rev. B.

[64] Ribeiro H.B., Pimenta M.A., de Matos C.J.S., Moreira R.L., Rodin A.S., Zapata J.D., de Souza E.A.T., Castro Neto A.H. (2015). Unusual Angular Dependence of the Raman Response in Black Phosphorus. ACS Nano.

[65] Strach T., Brunen J., Lederle B., Zegenhagen J., Cardona M. (1998). Determination of the phase difference between the Raman tensor elements of the A1g-like phonons in SmBa2Cu3O7−δ. Phys. Rev. B.

[66] Kranert C., Sturm C., Schmidt-Grund R., Grundmann M. (2016). Raman Tensor Formalism for Optically Anisotropic Crystals. Phys. Rev. Lett..

[67] Lei Z., Chen B., Koo Y.-M., MacFarlane D.R. (2017). Introduction: Ionic Liquids. Chem. Rev..

[68] Ferdeghini C., Mezzetta A., D’Andrea F., Pomelli C.S., Guazzelli L., Guglielmero L. (2022). The Structure–Property Relationship of Pyrrolidinium and Piperidinium-Based Bromide Organic Materials. Materials.

[69] Leys J., Wübbenhorst M., Menon C.P., Rajesh R., Thoen J., Glorieux C. (2008). Temperature dependence of the electrical conductivity of imidazolium ionic liquids. J. Chem. Phys..

[70] Macutkevic J., Banys J., Kania A. (2022). Electrical Conductivity and Dielectric Relaxation in Ag1−xLixNbO3. Crystals.

[71] Jonscher A.K. (1999). Dielectric Relaxation in Solids. J. Phys. D Appl. Phys..

[72] Enneffatia M., Rasheed M., Louatia B., Guidaraa K., Shihab S., Barillé R. (2021). Investigation of structural, morphology, optical properties and electrical transport conduction of Li0.25Na0.75CdVO4 compound. J. Phys. Conf. Ser..

[73] Totz J., Michel D., Bany J., Klopperpieper A. (1998). Conductivity processes in deuterated betaine phosphate1−xbetaine phosphitex mixed crystals. J. Phys. Condens. Matter.

[74] Balashova E.V., Svinarev F.B., Ankudinov A.V., Pankova G.A., Lityagin G.A., Kunkel T.S., Krichevtsov B.B. (2019). Polarization switching, dielectric, structural and elastic properties of 2-Methylbenzimidazole crystals and films. Ferroelectrics.

