# Supplementary material for: Crystal Structure, Raman, FTIR, UV-Vis Absorption, Photoluminescence Spectroscopy, TG–DSC and Dielectric Properties of New Semiorganic Crystals of 2-Methylbenzimidazolium Perchlorate
Source: Materials (Basel). 2023 Feb 28;16(5):1994. doi: 10.3390/ma16051994 (PMC10004103; doi:10.3390/ma16051994)
Supplement: Supplementary file 1 [file materials-16-01994-s001.zip › materials-2213976-supplementary.pdf]

Supplementary Materials

Crystal Structure, Raman, FTIR, UV-Vis Absorption, Photoluminescence Spectroscopy, TG–DSC and Dielectric Properties of New Semiorganic Crystals of 2-Methylbenzimidazolium Perchlorate

Elena Balashova [1,*], Andrey Zolotarev [2], Aleksandr A. Levin [1], Valery Davydov [1], Sergey Pavlov [1], Alexander Smirnov [1], Anatoly Starukhin [1], Boris Krichevtsov [1], Hongjun Zhang [3], Fangzhe Li [4], Huijiadai Luo [4] and Hua Ke [4]

[1] Ioffe Institute, Politechnicheskaya 26, 194021 Saint Petersburg, Russia
[2] Institute of Earth Sciences, Saint Petersburg State University, Universitetskaya nab. 7/9, 199034 Saint Petersburg, Russia
[3] School of Instrument Science and Engineering, Harbin Institute of Technology, Harbin 150080, China
[4] School of Materials Sciences and Engineering, Harbin Institute of Technology, Harbin 150080, China
* Correspondence: balashova@mail.ioffe.ru

S.I Details of powder XRD experiment, XRD line profile analysis, and crystal structure refinement

The following details of the X-ray diffraction (XRD) measurements not indicated in the main text of the paper should be mentioned. In order to reduce the effects of the preferred orientation of the crystallites, during the measurements the powder sample was rotated around the goniometer axis perpendicular to the sample holder. The correction shifts (zero shift $\Delta 2\theta_{zero}$ and displacement $\Delta 2\theta_{displ}$) of the XRD pattern were determined in the additional measurements of the sample powder in the mixture with certified standard silicon powder Si640f (NIST, Gaithersburg, MD, USA).

The obtained correction shifts were applied to the observed Bragg angles of the reflections as

$$2\theta_B = 2\theta_B^{obs} + \Delta 2\theta_0 + \Delta 2\theta_{displ}\cdot\cos(\theta_B^{obs}).$$

The corrected Bragg angles $2\theta_B$ of all observed reflections *hkl* were used for calculation of unit cell parameters of the MBI-perchlorate (MBI·$HClO_4$) using program *Celsiz* [1] and for Williamson-Hall plot (WHP) [2] and size-strain plot (SSP) [3]evaluation of microstructure parameters (mean crystallite size *D* and absolute mean values of microstrain ε_s in them) utilizing program *SizeCr* [4]. Program *SizeCr* calculated the criterium of the profile type $FWHM/B_{int} = 0.79(8)$, which is typical for pseudo-Voigt (pV) reflections ($0.637 < FWHM/B_{int} < 0.939$) [5], where *FWHM* and B_{int} are the observed full width at half maximum (FWHM) and integral width of the reflections. The values of $FWHM_{corr}$ of the FWHMs of the observed reflections, corrected for instrumental broadening in accordance with the pV type of reflections [2], [4], were used for calculations of WHP and SSP. Coefficient $K_{Scherrer} = 0.94$ of the Scherrer equation was used.

Results of WHP and SSP calculations for observed pV reflections of MBI·$HClO_4$ are shown in Figure S1. The determination coefficient R_{cod} (see [4], [6] for definition) is higher for SSP, reflecting higher accuracy of the SSP technique [3]. Both the WHP and SSP graphs show a view typical of the absence of microstrains ($\varepsilon_s = 0$), i.e., coefficient of the approximating lines $Y = A + B\cdot X$ of the WHP and SSP methods $B < 0$ for WHP [2], [5], [4] and $A < 0$ in case of SSP [3], [4]. In this case of microstrain absence ($\varepsilon_s = 0$), the program *SizeCr*

calculates the individual values of crystallite size D_{hkl} for each observed reflection *hkl* and a mean value of *D* by root-mean-square averaging of the D_{hkl} set.

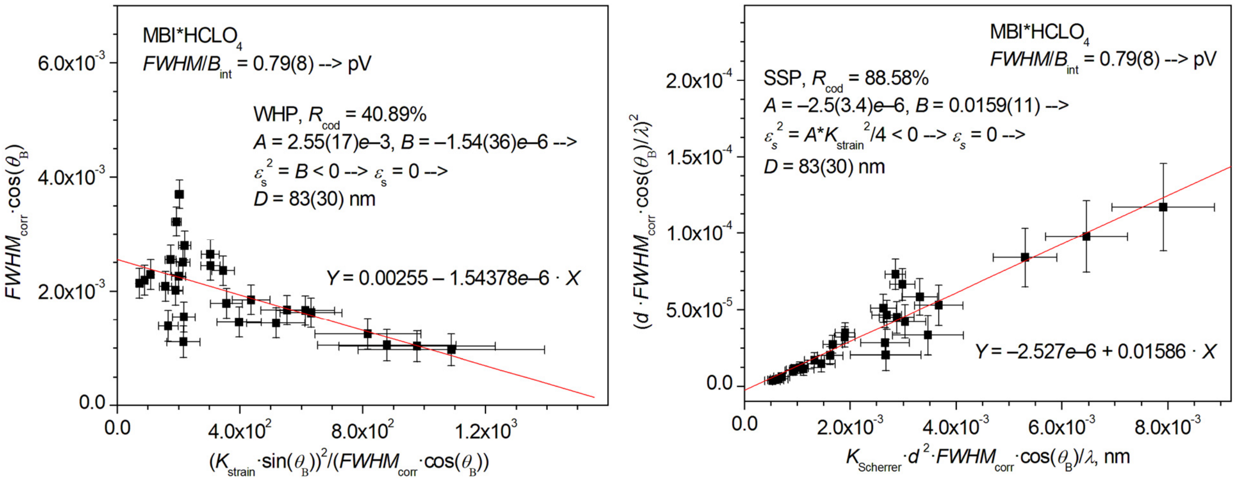

(**a**) (**b**)

Figure S1. (**a**) WHP and (**b**) SSP graphs for MBI-perchlorate. The coefficients $K_{Scherrer}$ = 0.94 and K_{strain} = 4 are used. $FWHM_{corr}$ is the FWHM of the observed reflection with Miller indices *hkl* which is corrected for instrumental broadening in accordance with the pV type of the reflection; *d* is the interplanar distance corresponding to the *hkl* reflection; θ_B is half of the Bragg angle $2\theta_B$ of the *hkl* reflection after applying the angular corrections ($\Delta 2\theta_{zero}$ and $\Delta 2\theta_{displ}$); λ is the wavelength of Cu-$K_{\alpha 1}$ radiation (λ = 1.540598 Å) after substraction of the Cu-$K_{\alpha 2}$ contribution; B_{int} is the integral width of the *hkl* reflection, $B_{int} = I_{int}/I_{max}$, where I_{int} and I_{max} are integral and maximum intensities of the reflection, respectively; R_{cod} is the determination coefficient. The formulas $Y = A + B \cdot X$ are also shown for the approximating WHP and SSP lines.

The following details of Le Bail (LB) and Rietveld refinements and fitting by program *TOPAS* [7] should be added to their description. The emission spectrum of the Cu-K_α radiation was described in frames of the Berger model of the five spectral lines [8] as it is recommended for the radiation filtered by Ni filter [7]. Conventional weight scheme $w_i = 1/I_i$, where I_i is the intensity value at diffraction angle $2\theta_i$ of the *i*-th step of the XRD pattern, was used for the LB and Rietveld refinements. The background of the XRD pattern was described by Chebyshev's polynomial of 7th order with a contribution of hyperbolic function for small diffraction angles ($2\theta < 10^{\circ}$). Instrumental broadening of XRD reflections was modeled based on the geometry of the diffractometer and the slits used, setting the type of reflections from the first principles (FP reflection type in *TOPAS* or fundamental approach in Ref. [9]). The remaining broadening of the reflections gave the mean size of the crystallites, which was refined in LB and Rietveld fitting, utilizing the double Voigt approach [10]. In order to compare with the results of WHP and SSP, the *LVol-FWHM* quantity calculated in *TOPAS* from the simulated FWHM using the Scherrer coefficient $K_{Scherrer}$ = 0.94 (same as in WHP/SSP calculations) was considered as the mean size *D* of the crystallites. Based on the results of WHP and SSP, it was assumed that there was no contribution of microstrains to the broadening of reflections.

The coefficients $m_{e.s.d.}$ for correction of the estimated standard deviations (e.s.d.s) underestimated due to serial correlations during LB and Rietveld refinement [11] were evaluated using program *RietESD* [12]. The agreement factors (weight profile factor R_{wp}, profile factor R_p, Bragg factor R_B, see, for example, [13] for definition of all agreement factors) were calculated using the Rietveld program *TOPAS* during the fitting course and checked by program *RietESD*. For calculation of the background-corrected analogues of the weight profile (cR_{wp}) and profile (cR_p) factors the program *RietESD* was

utilized (in case of use of hyperbolic function for description of a contribution to background, *TOPAS* gives wrong values of cR_{wp} and cR_p, see an example in Ref. [12]).

Results of LB fitting are presented in Figure S2.

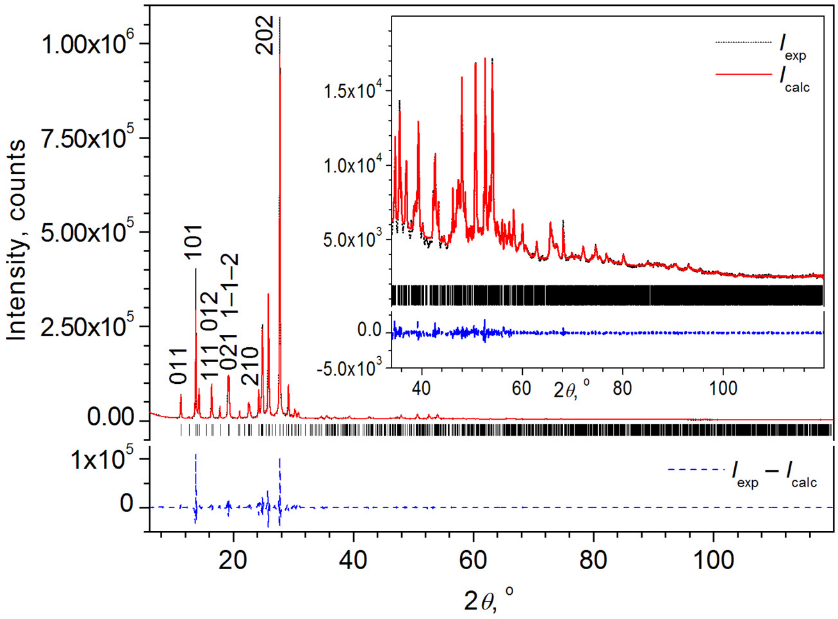

Figure S2. Final graphical results of LB fitting of the MBI-perchlorate (space group $P2_1/n$ (14)) powder XRD pattern. The Bragg angle positions of all allowed reflections are shown by bar diagram. Agreement factors R_{wp} = 10.83%, R_p = 7.23%, cR_{wp} = 15.92%, cR_p = 12.92%. E.s.d.s's-correcting coefficient $m_{e.s.d.}$ = 3.527. Unit cell parameters a = 7.9177(2) Å, b = 9.5116(1) Å, c = 12.6576(4) Å, β = 95.421(2)°, unit cell volume V_{cell} = 992.88(4) Å^3. Mean crystallite size D = 101(1) nm.

The Rietveld refinement of MBI·$HClO_4$ was carried out with atomic coordinates obtained for Sample 1 in single-crystal XRD analysis (Table S1) and results of LB fitting (unit cell parameters parameters, crystallite size and background parameters) as the initial model. Taking into account the effect of preferential orientation in the framework of the March-Dollase (MD) [14] model along two crystallographic directions ([101] and [111]), as allowed in *TOPAS*, gave a decrease in the weight profile factor R_{wp} by 26.27%. Taking into account the contribution of the other directions of preferential orientation within the framework of the 8th-order spherical harmonics model [15] led to a decrease in R_{wp} by another 7.37%. The inclusion of the overall temperature factor $B_{iso}^{overall}$ of all atoms and the coordinates of the atoms in the refinement led to a decrease in R_{wp} by 1.47%. In order to avoid correlations during the refinement of the structure, the parameters of the preferred orientation were refined in different cycles with temperature factors and atomic coordinates of atoms. During the Rietveld refinement of the coordinates of atoms, restrictions were imposed on the distances in polyhedra and groups (for Cl − O distances were restricted to vary between 1.39 and 1.49 Å, C − C 1.3 − 1.5 Å, C − N 1.28 − 1.45 Å, C − H 0.8 − 1.1 Å, and N − H 0.8 − 1.1 Å). At the last stage, the isotropic temperature factors overall for non-hydrogen atoms of the same sort (Cl, O, C and N) were refined. Similar to single crystal XRD, the temperature isotropic factors of the H atoms were also fixed at values equal to 1.2·U_{iso} for C_6H_6 and $C_3(NH)_2$ groups and 1.5·U_{iso} for CH_3 tetrahedron and were not refined (here U_{iso} is an isotropic temperature atomic parameter of a non-hydrogen atom nearest to the H atom). The weight profile factor decreased by another 0.05%, reaching the final value of R_{wp} = 12.67%. The remaining factors of agreement and graphical results of fitting are given in the main text of the article.

S.II. Crystal structure parameters of MBI-perchlorate according to single crystal and powder X-ray diffraction data

Crystal structure parameters of MBI-perchlorate (MBI·$HClO_4$) obtained in refinement of the structure model using single-crystal and powder XRD data are summarized in Tables S1 and S2. E.s.d.s obtained in in the refinements are shown in round brackets after the refined values of the parameters. Tables S3 and S4 presents the selected bond lengths (interatomic distances) and angles calculated using the structure data of the Table S1. The values of e.s.d.s of interatomic distances and angles between the specified atoms, indicated in round parentheses in Tables S3 and S4 and in the following text and tables, are calculated based on the values of e.s.d.s of the corresponding coordinates of the atoms (Table S1). The mean values of the interatomic distances and angles shown in angle brackets and their e.s.d.s are calculated using the root-mean-square (rms) averaging of individual values of these parameters. An increase of unit cell volume V_{cell} and corresponding decrease of calculated theoretical density ρ_{calc} of MBI- perchlorate in powder XRD in comparison to single-crystal one (Table S1) is due to higher temperature of measurements T_{meas} in case of XRD powder data collection.

Table S1. Results of refinement of the MBI-perchlorate structure (space group $P2_1/n$ (14)) using single crystal and powder XRD data (for Sample 1). Atomic numbers Z_{at}, relative coordinates of atoms (x/a, y/b, z/c), and their equivalent/isotropic temperature factors $U_{eq/iso}$ are shown. In the structure, all atoms are placed in a general Wyckoff position 4(e). Monoclinic unit cell parameters a, b, c, β and unit cell volume V_{cell}, calculated mass density ρ_{calc}, and temperature T_{meas} of data collection are indicated also.

Atom	Z_{at}	x/a	y/b	z/c	$U_{eq/iso}$, Å^2 [a]	x/a	y/b	z/c	$U_{iso}^{overall}$, Å^2 [b]
		Single crystal [c]: a = 7.6940(2) Å, b = 9.9774(2) Å, c = 12.4450(4) Å, β = 94.674(3)°, V_{cell} = 952.17(4) Å^3, ρ_{calc} = 1.6236(1) g/cm^3, T_{meas} = 100±2 K				Powder [d]: a = 7.9123(1) Å, b = 9.9617(5) Å, c = 12.6787(3) Å, β = 95.404(1)°, V_{cell} = 994.89(6) Å^3, ρ_{calc} = 1.3339(1) g/cm^3, T_{meas} = 313±1 K			
Cl01	17	0.58576(7)	0.56581(5)	0.78772(4)	0.0270(2)	0.5868(4)	0.57091(6)	0.7886(3)	0.091(2)
O002	8	0.7491(3)	0.5916(2)	0.84629(16)	0.0395(5)	0.7441(8)	0.5865(13)	0.8482(7)	0.098(2)
O003	8	0.5297(3)	0.68346(19)	0.72554(15)	0.0383(5)	0.5266(7)	0.6826(13)	0.7260(6)	0.098
O004	8	0.6033(3)	0.45642(19)	0.71356(15)	0.0376(5)	0.6025(8)	0.4630(12)	0.7099(6)	0.098
O005	8	0.4565(3)	0.5353(2)	0.86061(16)	0.0414(5)	0.4598(7)	0.5383(11)	0.8571(7)	0.098
N006	7	0.7888(3)	0.6837(2)	0.37251(16)	0.0269(5)	0.7873(9)	0.6853(14)	0.3758(8)	0.084(4)
H006	1	0.81718	0.74040	0.32496	0.032	0.8180(65)	0.7338(109)	0.3176(59)	0.100
N007	7	0.6760(2)	0.5999(2)	0.51101(16)	0.0269(5)	0.6779(9)	0.6024(15)	0.5073(8)	0.084
H007	1	0.61949	0.59375	0.56763	0.032	0.6154(64)	0.5843(113)	0.5719(58)	0.100
C008	6	0.7599(3)	0.4941(2)	0.46378(19)	0.0272(5)	0.7608(11)	0.4902(17)	0.4644(10)	0.076(1)
C009	6	0.8325(3)	0.5480(2)	0.37445(19)	0.0263(5)	0.8374(11)	0.5473(16)	0.3704(10)	0.076
C00A	6	0.9313(3)	0.4713(3)	0.3091(2)	0.0310(5)	0.9306(11)	0.4740(17)	0.3106(10)	0.076
H00A	1	0.98116	0.50761	0.25006	0.037	0.9797(58)	0.5147(108)	0.2508(55)	0.091
C00B	6	0.6315(3)	0.8456(3)	0.4840(2)	0.0333(6)	0.6391(11)	0.8417(17)	0.4852(10)	0.076
H00B	1	0.72161	0.91102	0.47761	0.05	0.7247(62)	0.9106(109)	0.4768(60)	0.114
H00C	1	0.59889	0.8443	0.5568	0.05	0.6026(64)	0.8437(105)	0.5568(57)	0.114
H00D	1	0.53182	0.86844	0.43601	0.05	0.5297(84)	0.8674(102)	0.4353(77)	0.114
C00C	6	0.8778(3)	0.2830(3)	0.4270(2)	0.0339(6)	0.8792(11)	0.2856(18)	0.4244(9)	0.076
H00E	1	0.89429	0.19256	0.44263	0.041	0.8939(65)	0.1898(100)	0.4449(56)	0.091
C00D	6	0.6964(3)	0.7119(2)	0.45548(19)	0.0271(5)	0.6969(11)	0.7149(18)	0.4543(10)	0.076

C00E	6	0.7819(3)	0.3601(3)	0.4926(2)	0.0306(5)	0.7800(11)	0.3598(18)	0.4910(9)	0.076
H00F	1	0.73458	0.3245	0.55278	0.037	0.7315(64)	0.3233(108)	0.5555(61)	0.091
C00F	6	0.9513(3)	0.3379(3)	0.3368(2)	0.0335(6)	0.9485(11)	0.3392(19)	0.3409(10)	0.076
H00G	1	1.01531	0.2826	0.29467	0.04	1.0139	0.2821	0.3016	0.091

[a] Equivalent isotropic temperature factors U_{eq} are shown for non-hydrogen atoms refined in an approximation of anisotropic temperature factor. For atoms of H, the isotropic temperature factors U_{iso} are shown (see note [c]).
[b] Overall isotropic temperature factor for atoms of one sort (for H atoms see note [d]). The isotropic temperature factors $B_{iso}^{overall}$ from the output of the Rietveld program *TOPAS*, are transformed to $U_{iso}^{overall}$ as $U_{iso}^{overall} = B_{iso}^{overall}/(8\pi^2)$. E.s.d.s are shown for refined values only.
[c] The coordinates of hydrogen atoms were calculated from geometric consideration of the corresponding rigid groups (CH, NH, CH_3) and were not refined. Their temperature isotropic factors were also fixed at values equal to $1.2 \cdot U_{eq}$ for CH and NH groups and $1.5 \cdot U_{eq}$ for CH_3 and were not refined (here U_{eq} is an equivalent isotropic temperature atomic parameter of a non-hydrogen atom of the corresponding group). All non-refined parameters are given without e.s.d.s.
[d] A restriction on the values of the distances M-H in the nearest coordination sphere of M atoms (M = N, C) was imposed during the Rietveld refinement of the coordinates of hydrogen atoms, assuming that these distances cannot be less than 0.80 Å and more than 1.15 Å. Similar to single crystal XRD, the temperature isotropic factors of the H atoms were also fixed at values equal to $1.2 \cdot U_{iso}$ for C_6H_6 and $C_3(NH)_2$ groups and $1.5 \cdot U_{iso}$ for CH_3 and were not refined (here U_{iso} is an isotropic temperature atomic parameter of a non-hydrogen atom of the corresponding group). All non-refined parameters are given without e.s.d.s.

Symmetry codes of space group $P2_1/n$ (14) ($P\ 1\ 2_1/n\ 1$ (14) in the whole designations):

x, *y*, *z*
-*x*+1/2, *y*+1/2, -*z*+1/2
-*x*, -*y*, -*z*
x-1/2, -*y*-1/2, *z*-1/2

For the atoms refined in an approximation of anisotropic temperature factors, the equivalent isotropic factor U_{eq} is calculated as 1/3 of the trace of the orthogonalized $\mathbf{U}^{ij}$ tensor.

Table S2. Anisotropic atomic displacement parameters U^{ij} for the MBI·$HClO_4$ single crystal Sample 1 (Table S1).

Atom	U^{11}, Å^2	U^{22}, Å^2	U^{33}, Å^2	U^{23}, Å	U^{13}, Å^2	U^{12}, Å^2
Cl01	0.0302(4)	0.0249(4)	0.0267(4)	0.0004(2)	0.0075(2)	0.0011(2)
O002	0.0382(11)	0.0417(11)	0.0381(10)	-0.0047(8)	-0.0008(8)	0.0060(8)
O003	0.0491(11)	0.0325(10)	0.0336(10)	0.0090(8)	0.0053(8)	0.0077(8)
O004	0.0475(11)	0.0313(10)	0.0356(10)	-0.0098(8)	0.0133(9)	-0.0017(8)
O005	0.0500(12)	0.0348(10)	0.0433(11)	-0.0008(9)	0.0265(9)	-0.0018(8)
N006	0.0286(10)	0.0248(10)	0.0279(10)	0.0023(8)	0.0066(8)	-0.0008(8)
N007	0.0269(10)	0.0264(10)	0.0280(10)	0.0008(8)	0.0062(8)	-0.0003(8)
C008	0.0257(11)	0.0270(12)	0.0287(11)	-0.0001(10)	0.0008(9)	-0.0017(9)
C009	0.0256(11)	0.0277(12)	0.0255(12)	-0.0018(9)	0.0018(9)	-0.0019(9)
C00A	0.0289(12)	0.0354(13)	0.0289(12)	-0.0033(10)	0.0042(10)	-0.0011(10)
C00B	0.0351(14)	0.0270(13)	0.0386(14)	-0.0013(10)	0.0079(11)	0.0018(10)
C00C	0.0340(13)	0.0229(12)	0.0435(14)	-0.0030(11)	-0.0046(11)	0.0016(10)
C00D	0.0248(11)	0.0271(12)	0.0293(12)	-0.0012(9)	0.0017(9)	0.0003(9)
C00E	0.0304(12)	0.0283(12)	0.0329(13)	0.0037(10)	0.0009(10)	-0.0030(10)
C00F	0.0299(12)	0.0341(14)	0.0363(14)	-0.0096(11)	0.0013(10)	0.0045(10)

Table S3. Bond angles (°) in MBI-perchlorate structure according to results of structure refinement using single crystal and powder XRD data (Table S1) for Sample 1. Temperature T_{meas} of data collection is indicated.

**Bond angle** [a]	**Single crystal, T_{meas} = 100±2 K**	**Powder, T_{meas} = 313±1 K**
O002 – Cl01 – O003	109.74(12)	116.6(5)
O002 – Cl01 – O004	109.85(12)	108.5(6)
O002 – Cl01 – O005	110.09(13)	109.7(9)
O004 – Cl01 – O003	108.05(11)	103.6(7)
O005 – Cl01 – O003	108.55(12)	107.5(6)
O005 – Cl01 – O004	110.53(12)	110.7(5)
<O – Cl – O> in ClO_4	109.5(1.0)	109.4(4.3)
C009 – N006 – H006	125.2	111.4(6.2)
C00D – N006 – H006	125.2	133.2(4.2)
C008 – N007 – H007	125.1	116.5(4.1)
C00D – N007 – H007	125.1	130.5(4.4)
<C – N – H> in $C_3(NH)_2$	125.15(6)	122.9(10.6)
C00D – N006 – C009	109.6(2)	115.1(1.1)
C00D – N007 – C008	109.8(2)	113.0(1.1)
<C – N – C> in $C_3(NH)_2$	109.7(1)	114.1(1.2)
N007 – C00D – N006	108.6(2)	106.9(9)
N – C – N in $C_3(NH)_2$	108.6(2)	106.9(9)
C009 – C008 – N007	105.9(2)	103.9(9)
C008 – C009 – N006	106.1(2)	101.1(1.0)
<C – C – N> in $C_3(NH)_2$	106.0(1)	102.5(2.0)
C00E – C008 – N007	132.1(2)	134.7(1.1)
C00A – C009 – N006	131.9(2)	136.5(1.1)
<C – C – N> between C_6H_6 and $C_3(NH)_2$	132.0(1)	135.6(1.3)
N006 – C00D – C00B	126.1(2)	128.7(1.0)
N007 – C00D – C00B	125.3(2)	124.2(1.1)
<N – C – C> between in $C_3(NH)_2$ and C_6H_6	125.7(6)	126.5(3.2)
C00E – C008 – C009	121.9(2)	121.4(1.1)
C00A – C009 – C008	122.0(2)	122.3(9)
C00E – C00C – C00F	121.6(2)	123.5(9)
C00A – C00F – C00C	122.0(2)	123.9(1.2)
<C – C – C>$_1$ in C_6H_6	121.9(2)	122.8(1.1)
C00F – C00A – C009	116.1(2)	114.8(1.2)
C00C – C00E – C008	116.3(2)	114.2(1.2)
<C – C – C>$_2$ in C_6H_6	116.2(1)	114.5(4)
<C – C – C>$_{all}$ in C_6H_6	120.0(2.9)	120.0(4.4)
C009 – C00A – H00A	121.9	120.1(4.2)
C00F – C00A – H00A	121.9	125.2(5.3)

C008 – C00E – H00F	121.8	120.9(4.8)
C00C – C00E – H00F	121.8	125.0(4.3)
<C – C – H>$_1$ in C_6H_6	121.85(6)	122.8(2.7)
C00E – C00C – H00E	119.2	113.8(6.5)
C00F – C00C – H00E	119.2	122.7(5.7)
C00A – C00F – H00G	119.0	118.8(4.3)
C00C – C00F – H00G	119.0	117.4(3.4)
<C – C – H>$_2$ in C_6H_6	119.1(1)	118.2(3.7)
<C – C – H>$_{all}$ in C_6H_6	120.5(1.5)	120.5(3.9)
H00B – C00B – H00C	109.5	110.8(8.1)
H00B – C00B – H00D	109.5	107.7(7.8)
H00C – C00B – H00D	109.5	104.7(6.1)
C00D – C00B – H00B	109.5	110.6(5.0)
C00D – C00B – H00C	109.5	114.0(4.9)
C00D – C00B – H00D	109.5	108.6(6.8)
<C – C – H> between $C_3(NH)_2$ and CH_3	109.5(0)	109.4(3.2)

[a] mean values of bond angles shown in angle parenthesis are the rms values for the specified groups

Table S4. Bond lengths (Å) in MBI·$HClO_4$ structure according to results of structure refinement using single crystal and powder XRD data (Table S1.1) for Sample 1. Temperature T_{meas} of data collection is indicated.

Bond [a]	Single crystal, T_{meas} = 100±2 K	Powder, T_{meas} = 313±1 K
Cl01 – O002	1.4240(20)	1.403(8)
Cl01 – O003	1.4520(18)	1.423(12)
Cl01 – O004	1.4428(18)	1.481(11)
Cl01 – O005	1.4320(18)	1.426(9)
<Cl – O> in ClO_4	1.438(12)	1.43(3)
N006 – H006	0.8600	0.93(9)
N007 – H007	0.8600	1.01(7)
<N – H> in $C_3(NH)_2$	0.8600(0)	0.97(6)
N006 – C009	1.394(3)	1.434(21)
N007 – C008	1.392(3)	1.429(20)
<N – C>$_1$ in $C_3(NH)_2$	1.393(1)	1.432(4)
N006 – C00D	1.330(3)	1.313(16)
N007 – C00D	1.330(3)	1.323(22)
<N – C>$_2$ in $C_3(NH)_2$	1.330(0)	1.318(7)
<N – C>$_{all}$ in $C_3(NH)_2$	1.36(4)	1.37(7)
C008 – C009	1.392(3)	1.499(19)
C008 – C00E	1.391(4)	1.347(24)
C009 – C00A	1.388(3)	1.326(19)
C00A – C00F	1.380(4)	1.400(25)
C00C – C00E	1.379(4)	1.415(19)
C00C – C00F	1.407(4)	1.347(19)

<C – C> in C_6H_6	1.39(1)	1.39(6)
C00A – H00A	0.9300	0.97(8)
C00C – H00E	0.9300	0.99(10)
C00E – H00F	0.9300	1.00(8)
C00F – H00G	0.9300	0.94(9)
<C – H> in C_6H_6	0.9300(0)	0.98(3)
C00B – C00D	1.478(3)	1.412(23)
C – C between $C_3(NH)_2$ and CH_3	1.478(3)	1.41(2)
C00B – H00B	0.9600	0.98(9)
C00B – H00C	0.9600	0.98(7)
C00B – H00D	0.9600	1.05(8)
<C – H> in CH_3	0.9600(0)	1.00(4)

[a] mean values of bonds shown in angle parenthesis are the rms values for the specified groups

S.III. Comparison of crystal structure parameters in different samples of MBI·$HClO_4$ single crystals.

Photos of the samples are shown in Figure S3

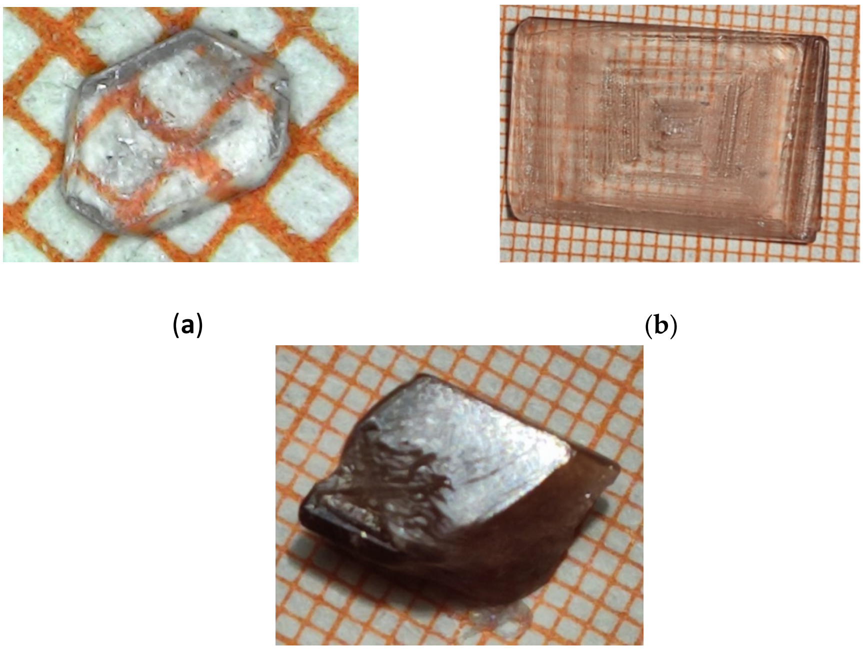

(**a**) (**b**) (**c**)

Figure S3. Optical images of (a) small transparent colorless plate Sample 1, (**b**) large Sample 2 with faint pink color, (**c**) thick bulk crystal (Sample 3) shown with distinct brown tint. The sizes of the grid steps in (**a**, **b**, **c**, **d**) are equal to 1 mm.

Table S5. Unit cell parameters and volume and selected experimental and structure refinement details of different samples of MBI·$HClO_4$ single crystals (temperature of data collection, T_{meas} = 100±2 K), obtained in single crystal structure refinements.

	**Sample 1**	**Sample 2**	**Sample 3**
Lattice parameters			
a, Å	7.6940(2)	7.69101(16)	7.69297(18)
b, Å	9.9774(2)	9.9944(2)	9.9898(2)
c, Å	12.4450(4)	12.4364(3)	12.4429(3)
β, deg	94.674(3)	94.619(2)	94.613(2).
Unit cell volume, V_{cell}, Å^3	952.17(4)	952.84(4)	953.16(4)
Theoretical density, ρ_{calc}, g/cm^3	1.6236(1)	1.6225(1)	1.6219(1)
h, *k*, *l* max	9, 10, 15	11, 15, 19	11, 15, 17
Reflections collected/Independent total/Independent $I >= 2\sigma(I)$]	5321/1812/1584	20577/3349/2829	18698/3400/2819
Final *R* indexes [Reflections $I >= 2\sigma(I)$]	R_1 = 0.0499, wR_2 = 0.1399	R_1 = 0.0382, wR_2 = 0.1045	R_1 = 0.0379, wR_2 = 0.0992
Final *R* indexes [Reflections all]	R_1 = 0.0553, wR_2 = 0.1477	R_1 = 0.0461, wR_2 = 0.1094	R_1 = 0.0479, wR_2 = 0.1053

Table S6. Selected bond lengths and angles of MBI molecule in different samples of MBI·$HClO_4$ single crystals (temperature of data collection, T_{meas} = 100±2 K).

**Crystal**	**Bond, Å C00D – N007**	**Bond, Å C00D – N006**	**Angle, ° N007 – C00D – N006**	**Angle, ° C00D – N007 – C008**	**Angle, ° C00D – N006 – C009**
**Sample 1**	1.330(3)	1.330(3)	108.6(2)	109.6(2)	109.8(2)
**Sample 2**	1.3331(16)	1.3333(16)	108.87(11)	109.42(10)	109.43(10)
**Sample 3**	1.3334(16)	1.3343(16)	108.69(11)	109.55(11)	109.57(11)
**Mean** [a]	1.3322(19)	1.3325(23)	108.72(14)	109.52(9)	109.60(19)

[a] round mean square (rms) values averaged over the values in samples 1, 2 and 3

Table S7. Selected bond lengths and angles of perchlorate tetrahedron ClO_4 in different samples of MBI-perchlorate single crystals (temperature of data collection, T_{meas} = 100±2 K)

	**Sample 1**	**Sample 2**	**Sample 3**	
Bond length, Å				<Cl – O>$_{samples}$ [a]
Cl01 – O002	1.4240(20)	1.4332(11)	1.4324(11)	1.4299(51)
Cl01 – O003	1.4520(18)	1.4520(10)	1.4534(11)	1.4527(8)
Cl01 – O004	1.4428(18)	1.4436(10)	1.4456(10)	1.4440(14)
Cl01 – O005	1.4320(18)	1.4355(10)	1.4362(11)	1.4346(23)
<Cl – O>[b]	1.438(12)	1.441(9)	1.442(9)	
Bond angles, °				<O – Cl – O>$_{samples}$[a]
O002 – Cl01 – O003	109.74(12)	109.56(7)	109.60(7)	109.63(9)
O002 – Cl01 – O004	109.85(12)	109.97(7)	110.04(7)	109.95(10)

O002 – Cl01 – O005	110.09(13)	109.99(7)	110.04(7)	110.04(5)
O004 – Cl01 – O003	108.05(11)	108.07(7)	108.09(7)	108.07(2)
O005 – Cl01 – O003	108.55(12)	108.70(7)	108.63(7)	108.63(8)
O005 – Cl01 – O004	110.53(12)	110.52(7)	110.42(7)	110.49(6)
<O – Cl – O>[b]	109.5(1.0)	109.5(9)	109.5(9)	

[a] rms values averaged over the values in samples 1, 2 and 3
[b] rms values for the ClO_4 tetrahedron of a sample

In the $C_3(NH)_2$ groups, the interatomic distances and angles are practically the same in different samples (Table S6), whereas they differ more in the ClO_4 tetrahedra (Table S7). In all samples, the largest bond length Cl01–O003 of the ClO_4 tetrahedra is almost the same. In contrast, the bond lengths Cl01–O002, Cl01–O004, Cl01–O005 in Samples 2 and 3 are greater than in Sample 1 (Table S7 and mean <Cl–O> values in the Table S7). The maximum deviation of the Cl01-O003 bond length from the average value <Cl–O> (rms interatomic distance Cl–O averaged over the ClO_4 tetrahedron of a sample) is 0.974% in the Sample 1, and 0.763% and 0.791% in the Samples 2 and 3, respectively. Corresponding mean deviations of the individual Cl–O distances in ClO_4 tetrahedron from the average values <Cl–O> are 0.675%, 0.467% and 0.527% for Samples 1, 2, and 3, respectively. The situation is similar with the angles between valence bonds of the O–Cl–O type. The maximum deviations from the average value <O–Cl–O> (rms-averaged over the ClO_4 tetrahedron of a sample) of the angle are 1.324% in the Sample 1, and 1.306% and 1.289% in the Samples 2 and 3 respectively. Also, corresponding mean deviations of the individual O–Cl–O angles in ClO_4 tetrahedron from the average values < O–Cl–O > are 0.702%, 0.650%, and 0.667% for Samples 1, 2, and 3, respectively. This shows that the ClO_4 tetrahedra are most strongly distorted in the Sample 1. In large colored crystals (Samples 2 and 3), they are closer to the shape of an ideal tetrahedron. The unit cell volume V in the Samples 2 and 3 is slightly larger than in Sample 1, which is mainly caused by larger values of parameters a and b and decrease of monoclinicity angle β. An increase in the cell volume V_{cell} and corresponding decrease in calculated XRD density ρ_{calc} (Table S5) is possible with the introduction of an impurity which size is commensurate with the voids in the unit cell. It can be assumed that impurities are formed as a result of the decomposition of perchloric acid over time.

S.IV. TG – DSC curves in MBI·$HClO_4$

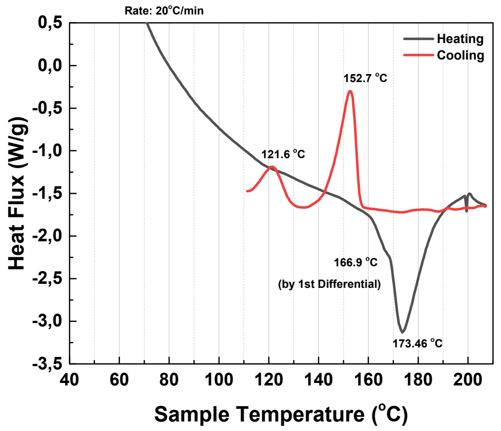

(a)

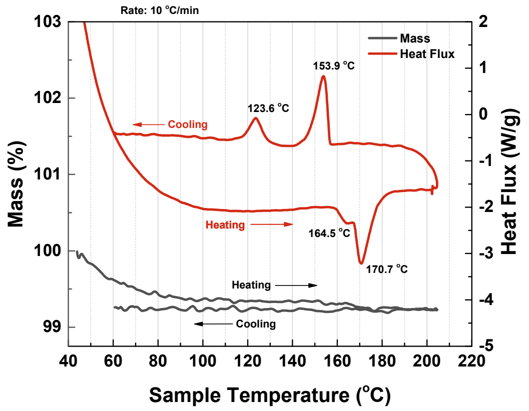

(b)

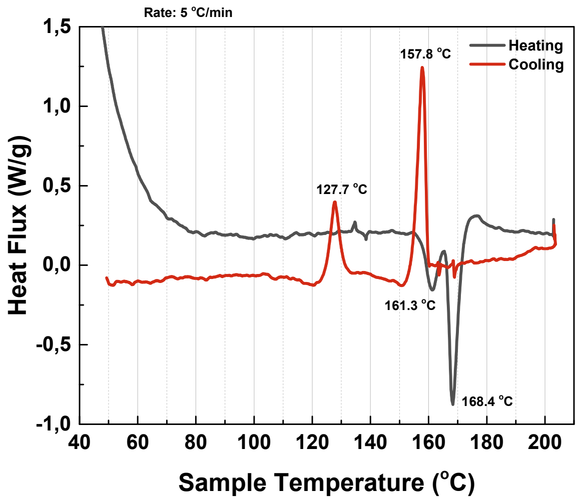

(c)

Figure S4. TG-DSC curves for heating and cooling of MBI-perchlorate crystals (Sample 1) measured at a rate of 20 °/min (**a**), 10 °/min (**b**), and 5 °/min (**c**).

References

[1] Maunders, C.; Etheridge, J.; Wright, N.; Whitfield, H. J. Structure and microstructure of hexagonal $Ba_3Ti_2RuO_9$ by electron diffraction and microscopy. *Acta Crystallogr. B* **2005**, *61*, 154–159. https://doi.org/10.1107/S0108768105001667

[2] Terlan, B.; Levin, A.A.; Börrnert, F.; Simon, F.; Oschatz, M.; Schmidt, M.; Cardoso-Gil, R.; Lorenz, T.; Baburin, I. A.; Joswig, J.- O.; Eychmüller, A. Effect of Surface Properties on the Microstructure, Thermal, and Colloidal Stability of VB_2 Nanoparticles. *Chem. Mater.* **2015**, *27*, 5106–5115. https://doi.org/10.1021/acs.chemmater.5b01856

[3] Terlan, B.; Levin, A. A.; Börrnert, F.; Zeisner, J.; Kataev, V.; Schmidt, M.; Eychmüller, A. A Size-Dependent Analysis of the Structural, Surface, Colloidal, and Thermal Properties of $Ti_{1-x}B_2$ (x = 0.03–0.08) Nanoparticles, *Eur. J. Inorg. Chem.* **2016**, *6*, 3460–3468. https://doi.org/10.1002/ejic.201600315

[4] Levin, A. A. Program *SizeCr* for calculation of the microstructure parameters from X-ray diffraction data. Preprint, **2022,** accessed on 5 June 2022. https://www.researchgate.net/profile/Alexander-Levin-6/research.

[5] Langford, J. I.; Cernik, R. J.; Louer, D. The Breadth and Shape of Instrumental Line Profiles in High-Resolution Powder Diffraction, J. Appl. Phys. 1991, 24, 913–919. https://doi.org/10.1107/S0021889891004375

[6] Balashova, E.; Levin, A. A.; Fokin, A.; Redkov, A.; Krichevtsov, B. Structural Properties and Dielectric Hysteresis of Molecular Organic Ferroelectric Grown from Different Solvents. *Crystals* **2021**, *11*, 1278. https://doi.org/10.3390/cryst11111278

[7] *TOPAS*, Version 5. Technical Reference; Brucker AXS: Karlsruhe, Germany, 2014.

[8] Berger, H. Study of the K alpha emission spectrum of copper, *X-ray Spectrom.* **1986**, 15, 241–243. https://doi.org/10.1002/xrs.1300150405

[9] Cheary, R. W.; Coelho, A. A. A fundamental parameters approach to X-ray line-profile fitting, J. Appl. Cryst. 1992, 25, 109–121. https://doi.org/10.1107/S0021889891010804

[10] Balzar, D. Voigt-function model in diffraction line-broadening analysis, in Snyder, R. L.; Fiala, J.; Bunge, H. J. (eds.) Defect and Microstructure Analysis by Diffraction, IUCr, Oxford Uni, Press, 1999, pp. 94–126. https://doi.org/10.1107/S1600576714001058

[11] Bérar, J.-F.; Lelann, P. J. E.S.D.'s and Estimated Probable Error Obtained in Rietveld Refinements with Local Correlations. *J. Appl. Crystallogr.* **1991**, *24*, 1–5. https://doi.org/10.1107/S0021889890008391

[12] Levin, A.A. Program *RietESD* for correction of estimated standard deviations obtained in Rietveld-refinement program. Preprint, **2022,** https://www.researchgate.net/profile/Alexander-Levin-6/research, accessed on 5 June 2022. https://doi.org/10.13140/RG.2.2.10562.04800

[13] Hill, R. J.; Fischer, R. X. Profile agreement indices in Rietveld and pattern-fitting analysis. J. Appl. Crystallogr. 1990, 23, 462–468. https://doi.org/10.1107/S0021889890006094

[14] Dollase, W.A. Correction of Intensities for Preferred Orientation in Powder Diffractometry: Application of the March Model. *J. Appl. Crystallogr*. **1986**, *19*, 267–272. https://doi.org/10.1107/S002188988860894

[15] Järvinen, M. Application of symmetrized harmonics expansion to correction of the preferred orientation effect. *J. Appl. Cryst*. **1993**, *26*, 525–531. https://doi.org/10.1107/S0021889893001219
